# A Mentor, Advisor, and Coach (MAC) Program to Enhance the Resident and Mentor Experience

**DOI:** 10.15766/mep_2374-8265.11005

**Published:** 2020-11-03

**Authors:** Dipal Patel, Donna Windish, Seonaid Hay

**Affiliations:** 1 PGY 4, Department of Nephrology, Internal Medicine, Yale University School of Medicine; 2 Associate Professor of Medicine (General Medicine); Director, Resident Research, Yale Primary Care Residency Program; Program Director, General Internal Medicine Medical Education Fellowship, Yale University School of Medicine; 3 Assistant Professor of Medicine (General Medicine); Associate Program Director, Internal Medicine Traditional Residency Program, Yale University School of Medicine

**Keywords:** Mentorship, Mentors, Residency, Internal Medicine, Advising, Coaching

## Abstract

**Introduction:**

While residents must meet standardized educational milestones to graduate, individualized mentorship and guidance can help them achieve personal and career goals. A novel mentor, advisor, and coach (MAC) program was created for residents of the Yale University Traditional Internal Medicine Residency Program to help them attain these goals.

**Methods:**

Internal medicine faculty were recruited into the MAC program and matched with residents, with each faculty paired with one to three mentees. A structured roadmap was used to guide program content (including topics of mentoring, advising, and coaching), and meetings were individualized to cater to the needs of residents. During the 2017–2018 academic year, online surveys and focus groups were used to obtain feedback from participants.

**Results:**

Survey responses were obtained from 50 of the 116 residents (43%) and 21 of the 49 MAC faculty (43%). Thirteen residents and five MAC faculty participated in in-person focus groups. Most participants (92% of interns, 83% of residents, and 95% of MAC faculty) felt the program was beneficial and should continue. Individualized relationships and meeting content were key to the program's success. Areas for improvement included clarification of the program's purpose and each party's responsibilities in scheduling meetings. MAC faculty also requested faculty development tools to help them meet expectations of being a MAC.

**Discussion:**

The MAC program provided a successful avenue for mentorship and guidance for residents. Central themes to enhance participants' experience were individualization and flexibility, mutual agreement of the ground rules, and enhanced communication from program leadership.

## Educational Objectives

By the end of this activity, learners will be able to:

1. Describe the importance of providing residents with access to confidential and personalized mentoring relationships.
2. Identify key features contributing to successful relationships between faculty and mentees of residency training programs.
3. Apply the materials provided to implement a mentor, advisor, and coach program at their own institutional training programs.

## Introduction

Medical residency is challenging in educational and personal development. Trainees must meet standardized educational milestones, as assessed by ACGME requirements. Residents also need to develop career goals and implement steps to achieve those goals, including networking within their field of interest, completing research projects, and applying for fellowships or jobs. These less standardized aspects of training may require individualized guidance.

Mentorship can entail several roles, including teaching, advising, coaching, and role modeling.^1^ Successful mentoring relationships provide emotional support and have mutually agreed goals between mentors and mentees.^2–4^ Participation in a mentorship program should promote development of both the mentee and mentor.^5^

As of 2016, residents of the Yale University Traditional Internal Medicine Residency program had associate program director (APD) advisors as their main source of mentorship. However, as a large program (116 residents), each APD is assigned over 30 residents to advise, leading to infrequent and sometimes perfunctory meetings. To address this, members of the program leadership created the mentor, advisor, and coach (MAC) program. The goals of the MAC program were to (1) identify trainee needs in achieving personal and career goals, and (2) pair trainees with faculty who could help them attain these goals in an individualized manner.

In this publication we describe the implementation of the MAC program and provide resources which can be utilized for the creation of similar programs at other institutions. While principles of mentoring and advising have been published by others,^6,7^ this program was unique in its flexible structure, with individualized relationships between residents and MAC faculty, often with variable emphasis on mentoring, advising, and/or coaching. It provides a framework for faculty involvement without significant contribution of time. We also present direct feedback from participants highlighting program successes and areas for improvement, both of which can be incorporated as the program becomes adapted across different training programs.

There is increasing evidence that mentee-driven relationships foster program success.^6,8,9^ The MAC program was designed to be flexible and individualized to meet the needs of residents as they unfold during residency training.

## Methods

### MAC Program Structure

During the 2015–2016 academic year, leadership of the Yale University Traditional Internal Medicine Residency Program created the MAC program. To limit time and effort required by each MAC faculty, we assigned each MAC faculty one resident per PGY class for a maximum of three mentees. Residents were assigned to this MAC faculty member for the full 3 years of training. We started our list of MAC faculty by first surveying residents to determine who they felt would be good for this role. From this list of potential MAC faculty, program leadership removed anyone in a fellowship director position to avoid conflicts of interest, as well as anyone who was deemed unsuitable (i.e., holding a program leadership position or being close to retirement). In the spring of 2016, we recruited 42 faculty within internal medicine to be MACs, and additional MAC faculty were recruited as needed in subsequent years.

To provide MACs with the skills needed to be comfortable and proficient in their new roles, the APDs hosted a 3-hour faculty development session (didactic, discussion, and question-and-answer sessions), which provided information on different aspects of the MAC role (Appendices A and B). General topics of discussion for each MAC-mentee meeting, referred to as roadmaps, were included in this presentation and subsequently distributed via email throughout the year. It was emphasized in training that MACs were not meant to serve as career or research mentors, but that they could help mentees find these additional mentors. MACs were also not expected to be the main source of support for fellowship applications, as program leadership takes on this role. Meetings were recorded and made available for MAC faculty who were unable to attend, and a faculty guide was created for MACs to refer back to as needed (Appendix C).

To pair MAC faculty with residents, we surveyed residents regarding their demographics, career interests (field of interest and type of career), and other considerations which might be important to them in finding a match (gender, sexual orientation, family status, etc.; Appendix D). Matches were made with the following order of importance: (1) self-identified special requests (i.e., pairing a resident who is a mother of young children with a faculty who is a mother of young children), (2) gender, (3) outpatient clinic site (to help facilitate meetings which mostly occur during ambulatory blocks), and (4) future career type of interest (i.e., clinician-educator with clinician-educator). Of MACs, 55% were subspecialists, and due to the potentially sensitive nature of discussions, we avoided pairing residents with faculty in their same field of interest (i.e., cardiology with cardiology). MAC faculty were not intended to be directly supervising their resident mentees at any point during training.

We emailed residents and MAC faculty at the beginning of the academic year, introduced them to each other and asked them to arrange a first meeting. We distributed a meet-and-greet questionnaire for residents to fill out and send to their respective MAC faculty before their first meeting, or to use as a framework for the first meeting (Appendix E). Every 2 months, we emailed all MAC faculty and mentees with the designated topic for upcoming meetings based on predetermined roadmaps (listed in Appendix A), though emails also stated that the mentee could instead speak to their MAC faculty about any pressing issues. We distributed worksheets on coaching (Appendix F) and mentoring (Appendix G) before meetings focusing on these topics. MAC faculty were also given access to their mentees' online evaluations to be used for coaching. While residents were asked to meet with their MACs at least once every ambulatory block (six times per year), meeting frequency was not strictly enforced and there were no consequences if pairs did not meet.

### Program Evaluation

Anonymous surveys were distributed online to the participating 116 categorical residents and 42 MAC faculty in December 2017 (during the program's second year). In the spring of 2018 (during the same academic year), all residents were invited to participate in one of two focus groups led by one of the authors (Dipal Patel) who used a list of questions to initiate discussion (Appendix H), with follow-up questions guided by participants' initial responses. A select number of MAC faculty were also invited to participate in a separate focus group led by Dipal Patel, using a separate list of questions (Appendix H). All focus group participants were guaranteed confidentiality, and discussions were transcribed by Dipal Patel without inclusion of identifying information.

Deidentified data from surveys and focus groups were independently reviewed by each author. Quantitative data were summarized using descriptive statistics and were reported as averages. Qualitative data (open-ended feedback) were reviewed by each author and was categorized into overarching themes, with inclusion of supporting quotes designated as coming from a resident or MAC faculty.

This research was conducted with a waiver from the Yale University Institutional Review Board.

## Results

**Figure 1.**
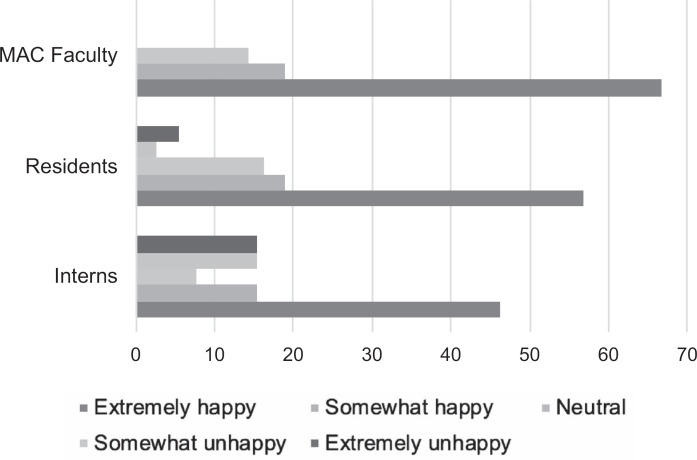
Satisfaction of interns (*N* = 13), residents (*N* = 37), and mentor, advisor, and coach (MAC) faculty (*N* = 21) with MAC faculty-mentee pairs.

**Figure 2.**
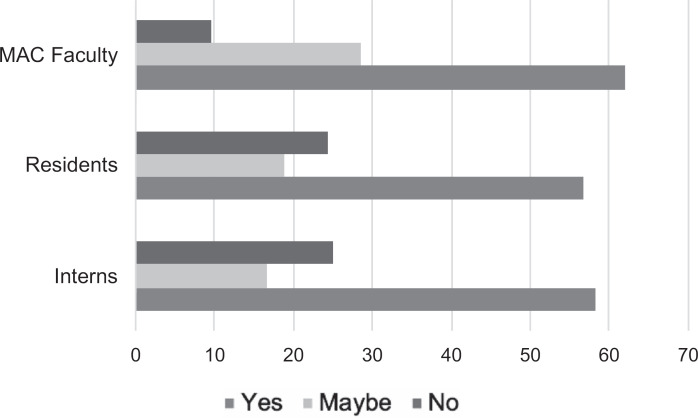
Survey responses from participants, asking about the ability of the mentor, advisor, and coach (MAC) program to enhance their experiences as an intern (*N* = 13), resident (*N* = 37), or MAC faculty (*N* = 21).

Every categorical resident in the program was assigned a MAC faculty. We received survey responses from 50 of the 116 categorical residents (43%) and 21 of the 49 MAC faculty (43%) who participated in the program during the 2017–2018 cycle. Most participants were satisfied with their pairings (Figure 1), and the majority (100% of interns, 90% of residents, and 95% of MAC faculty) were gender concordant (Table). Participants reported an average of 3.2 (*SD* 1.8) meetings per year. Use of meeting roadmaps was variable. While 57% of all participants felt that the program enhanced their experiences as an intern, resident, or faculty member (Figure 2), most participants (92% of interns, 83% of residents, and 95% of MAC faculty) felt that the program should continue (Table). Some residents who were less satisfied with pairings cited personality differences as a major contributor, and some participants felt that the program was redundant and not beneficial. However, residents commented that even though they may not have personally benefited from the program, it should remain in place as an option and to help others who may more consistently need it.

**Table.**
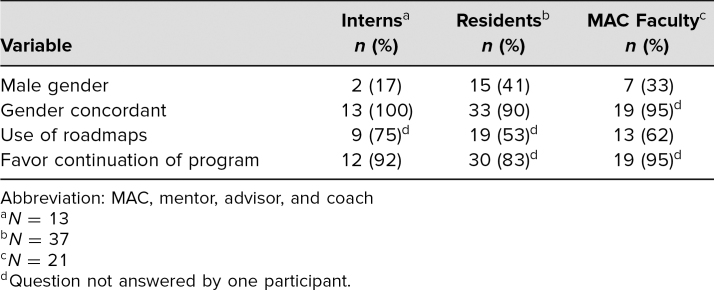
Descriptive Statistics Obtained From Survey Data

Two resident focus groups were held with a total of 13 participants (six PGY 1s, five PGY 2s, one PGY 3, and one PGY4 who had recently graduated and had participated in the program's first year). Five MAC faculty were asked to provide further qualitative feedback, with four participating in a MAC faculty focus group and one providing feedback via telephone. After reviewing data from surveys and focus groups, the following themes emerged as being central to the experiences of residents and MAC faculty.

### MAC Faculty-Mentee Pairings

Residents acknowledged the importance of having a MAC faculty who was not evaluating them, and with whom all discussions would be confidential. MAC faculty provided emotional support and helped residents navigate obstacles, in addition to understanding their individual motives. Residents valued having a MAC of the same gender, noting:

• “Compared to all other mentors, I feel that my MAC actually knows who I am and understands my motives, and is totally unassuming in the sense that she doesn't have specific expectations for me (just is there to support me and help out).”
• “I really appreciate having a MAC of the same gender. I feel that we are able to discuss the specific challenges for women in medicine and this would not be as easy if my MAC was male.”

Pairings could be enhanced by asking residents to identify any specific area of focus for the relationship (e.g., career guidance to match well for a competitive subspecialty vs. coaching through a known learning barrier). MAC faculty could also provide information on their own background so that mentees could easily identify shared experiences. Dissimilar personalities were reported as a major factor leading to dissatisfaction. One resident noted:

• “My MAC is a pleasant person and has made efforts to meet with me, but we don't mesh very well personality wise and I don't feel very comfortable sharing personal information with her…”

Residents requested MAC faculty to be familiar with timelines and steps required for fellowship and job applications. MAC faculty similarly reported a desire to be more prepared for questions pertaining to post-matriculation careers:

• “Fellowship is what's on their mind and it makes sense for us to be useful in that domain. I feel keen for more information. I haven't lived through fellowship. It's useful to know what's the state of the art right now so we can guide them…”

### Meeting Frequency

Scheduling conflicts and lack of time were significant barriers to meeting. If residents felt that they did not have anything to discuss with MAC faculty, they were less inclined to initiate a meeting. Furthermore, it was not always clear who was responsible for setting up meetings. An incentive to meet, such as a monetary allowance for coffee, might help encourage meetings. One MAC faculty stated:

• “It's super variable for interaction rates. Some email right away and those are really good relationships. Others it's like pulling teeth and I feel that if that's the way the interaction is, it's not valuable.”

Having MAC faculty and mentees work in the same clinic aligned schedules and encouraged interactions. Reminder emails before mentees' ambulatory blocks were helpful to initiate scheduling. An allotted conference time per ambulatory block could become a dedicated time for meetings. Scheduling in advance (at the end of the prior meeting) helped avoid delays in meeting.

Some MAC faculty arranged meetings with multiple mentees at one time. These meetings had the advantage of offering additional longitudinal mentoring (older residents could mentor interns in the same MAC family); however, larger meetings could be more difficult to schedule and may become less personal. One resident said:

• “In my medical school, we had med families of mentor pairings, e.g. year one with med siblings in year two, three, four and a faculty mentor leading the team. We could replicate that and merge the MAC program with the peer mentor program. Each resident could still meet with the MAC one on one but also have joint ‘family’ social events with PGY 1–3 and MAC.”

### Meeting Content/Structure

Meetings focused on issues mentees wished to discuss at that time, regardless of the outlined roadmap. Residents noted:

• “I have a good idea of what I need out of mentorship at this stage in my training, so I usually let that guide our meetings. I think it's a great structure for those who may not have as much clarity!”
• “We felt that we had better conversations when they were more organic and purely based on whatever we wanted to talk about then. I felt that this system felt more natural and was a lot more useful for me.”

### Scope of Program

Given the individual needs of each mentee at different points of training, it was felt that mentoring, advising, and coaching were all helpful and should remain in the scope of the program. MAC faculty requested additional resources, tools, and faculty development to define each of these roles, with one MAC faculty who observed:

• “I think we would want more tools about how to be the best MACs. … None of us were trained. If we can get faculty development in these areas it would make us better prepared for this role.”

MAC faculty were given access to mentees' online evaluations in an effort to coach mentees through any issues encountered. Some MAC faculty reported that coaching was the most difficult aspect of the MAC program, as it traditionally requires frequent meetings, and MAC faculty usually did not directly observe mentees in clinical scenarios. While online evaluations could help identify areas that residents were struggling in, they could also potentially detract from the MAC faculty-mentee relationship which is confidential and intentionally devoid of having any evaluative capacity. MAC faculty stated:

• “…I'm not sure what my role is to see evaluations—some are summative and I'm not sure if it's my role to go over that, or if it should be the chief residents or APDs. It may be lack of experience, or that we're not seeing them frequently enough for coaching, or we're not prepared. Mentoring and advising can be done with less frequent meeting. Program is not set up to be a good coach.”
• “I feel that it's helpful to see the evals because it provides some objective data about how the resident is telling you how they're doing. We're not great at self-reporting and self-assessing. Without the objective data we could be going around about things that don't exist. I have a mentee who is at the top half but not superstar, and it was helpful to match up the evals—in talking to her she seemed to be struggling but in looking through the evals it was much more positive…”

### Participant Satisfaction

The majority of participants felt that the program should continue:

• “I feel very blessed to be matched with my mentor. I feel warm, challenged, and more insightful after every visit with her. She meets me at my emotional point each time. She doesn't make me feel guilty for feeling emotional about residency. And she helps me problem solve. I leave the meeting with an actionable item. … That is so powerful!” (Resident)
• “It's refreshing to have a professional relationship with mentees that is also free from pressure to produce something clinically or academically.” (MAC faculty)

Both MAC faculty and residents reported that time constraints, difficulties with coordinating meetings, and having meetings that did not provide the support desired by residents were detrimental to their experiences:

• “It's so hard to find mentors to help you get through residency difficulties that are specific to an individual. Every resident and intern will have their own obstacles to overcome, and [it's] very unlikely that a randomly assigned mentor will have had the relevant experiences to genuinely be able to contribute insights that are helpful. In the absence of this type of tailored guidance, the MAC program becomes just another hoop that we need to jump through…” (Resident)
• “I think if the mentees had reached out without me having to remember, I would have enjoyed it even more.” (MAC faculty)

MAC faculty reported difficulty in not having protected time for this role. Some asked if there could be departmental recognition for their efforts, educational stipends for conferences and meetings, or if time used in this role could count towards promotions. Professional growth opportunities could help recruit and retain faculty.

## Discussion

Exposure to mentorship, advising, and coaching can guide development of residents. Individually addressing academic, learning, and social issues, while providing emotional support and encouragement, is key.^2,4,10^ Effective relationships have a mutual commitment offered by mentor and mentee, with initial meetings defining the relationship with agreed goals and responsibilities.^2,11,12^ Mentors should be adequately trained to fulfill the agreed roles.^13–16^ With this framework, both mentee and mentor can significantly benefit from the relationship.^3,5^

Individualization and flexibility of the MAC faculty-mentee relationship enhanced participants' experiences and improved participation. Residents appreciated MAC faculty asking about their individual motives, including issues of work-life balance and career goals. Meetings focusing on issues facing the resident at that particular time, regardless of the roadmap, were more helpful. MAC faculty could coach residents through these individual issues, setting up more frequent meetings as needed. Though a standard number of meetings was required to establish strong relationships, the timing and frequency of meetings was flexible to accommodate needs of each mentee.

Mutual agreement of the ground rules helped manage expectations. MAC faculty and residents are now asked to discuss mutual responsibility for coordination of meetings. MAC faculty are expected to set up the first meeting of each year, and to periodically reach out to mentees to check in on their progress. Otherwise, it was the responsibility of mentees to reach out to MAC faculty to schedule meetings throughout the year. A clear understanding of faculty roles was necessary. A MAC is not a career mentor or a letter writer, but can be a primary resource for referring mentees to other faculty who fulfilled these roles. Residents were aware that discussions with MAC faculty were confidential; however, program leadership can reach out to MAC faculty to discuss particular concerns about mentees. Finally, if a mentee found the relationship to be unsatisfying, he or she had the right to request a change in MAC faculty.

Communication from program leadership helped remind pairs to meet during ambulatory clinic blocks and included the suggested purpose of each meeting. Reminders emphasized that mentees could ask MAC faculty about individual issues they were facing, or discuss topics outlined in the roadmap. MAC faculty requested standardized resources to distribute to their mentees, in addition to information on fellowship and job applications, and a list of potential career and research mentors in each subspecialty field to be used when helping mentees establish these additional relationships. MAC faculty reported that retention and satisfaction could be improved with additional faculty development.

### Challenges

Several challenges were encountered in the implementation of the MAC program. While viewed as a strength by most participants, there were no repercussions if pairs did not meet or follow roadmaps, resulting in variable levels of participation. Despite efforts to match pairs successfully, some pairs inevitably did not mesh well, resulting in mentees being less interested in meeting with their MAC. While the program was designed to be tailored to meet the needs of each resident, it was clear that fulfilling the roles of a mentor, advisor, and coach was sometimes unattainable.

### Limitations

Feedback was elicited by a convenience sample and therefore has limitations in its generalizability. While some areas of discussion were felt to reach thematic saturation, others (specifically, feedback on access to online evaluations) were added at the end of focus group analysis and were not included in the initial survey, and did not reach saturation. Primary data were reviewed by all authors, but coding was not used and the information was instead informally summarized to construct the themes presented above. Finally, hard outcomes such as development of tangible skills or matriculation into fellowships or career pathways were not measured.

### Conclusions

A structured program offering individualized mentorship, advising, and coaching can enhance residents' experience during graduate medical education, and provide a valuable opportunity for faculty to become uniquely involved in residents' training. MAC programs should be flexible and tailored to the individual needs of each resident, and can be developed widely across residency training programs to assist residents in achieving personal and career goals. Faculty can also benefit from these gratifying interactions with relatively low amounts of time commitment. With additional cohorts within and across institutions, concrete outcome measures such as career satisfaction, wellness metrics, remediation rates, and overall satisfaction can be analyzed.

## Appendices

MAC Training Presentation.pptxMAC Training Facilitator Guide.docxMAC Faculty Guide.docxMAC Survey - Resident Pairings.docxMeet and Greet Questionnaire.docCoaching Worksheet.docxMentoring Worksheet.docxQuestions for Focus Groups.docx
All appendices are peer reviewed as integral parts of the Original Publication.
