# Supplementary material for: A Mentor, Advisor, and Coach (MAC) Program to Enhance the Resident and Mentor Experience
Source: MedEdPORTAL. 2020 Nov 3;16:11005. doi: 10.15766/mep_2374-8265.11005 (PMC7666835; doi:10.15766/mep_2374-8265.11005)
Supplement: Supplementary file 1 — MAC Training Presentation.pptxMAC Training Facilitator Guide.docxMAC Faculty Guide.docxMAC Survey - Resident Pairings.docxMeet and Greet Questionnaire.docCoaching Worksheet.docxMentoring Worksheet.docxQuestions for Focus Groups.docx [file mep_2374-8265.11005-s001.zip › A. MAC Training Presentation.pptx]

## Slide 1
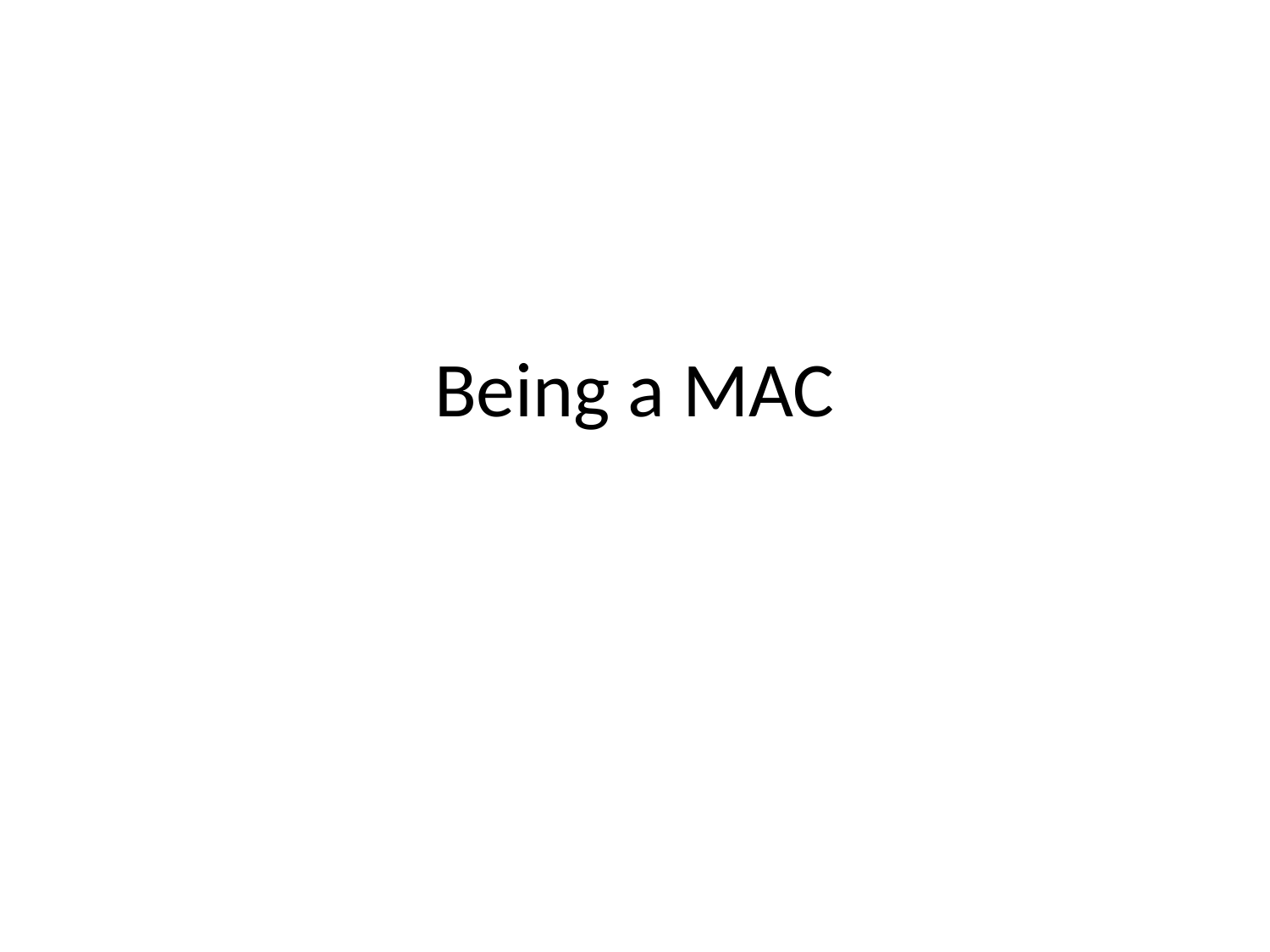

# Being a MAC

## Slide 2
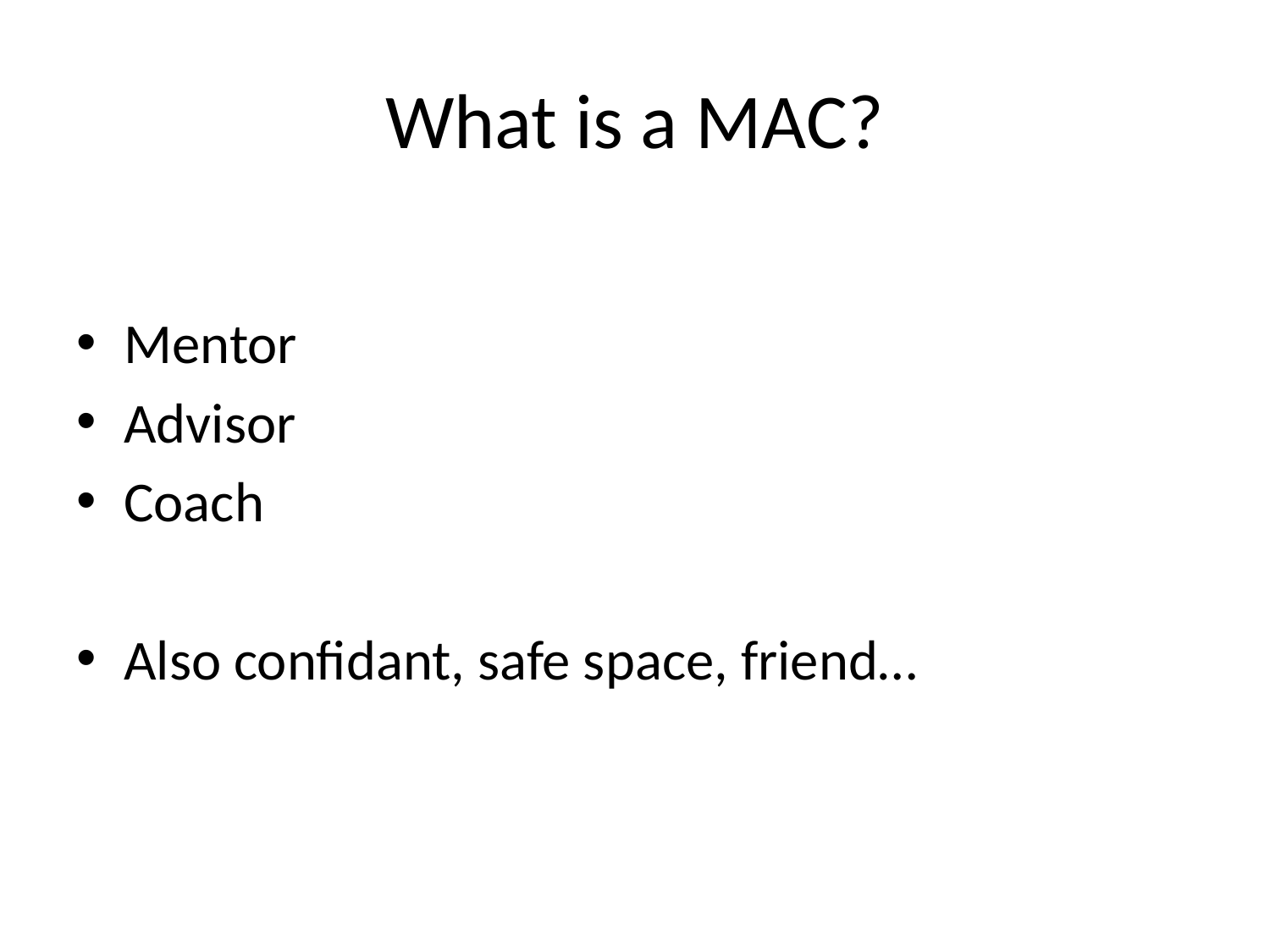

# What is a MAC?
Mentor
Advisor
Coach
Also confidant, safe space, friend…

## Slide 3
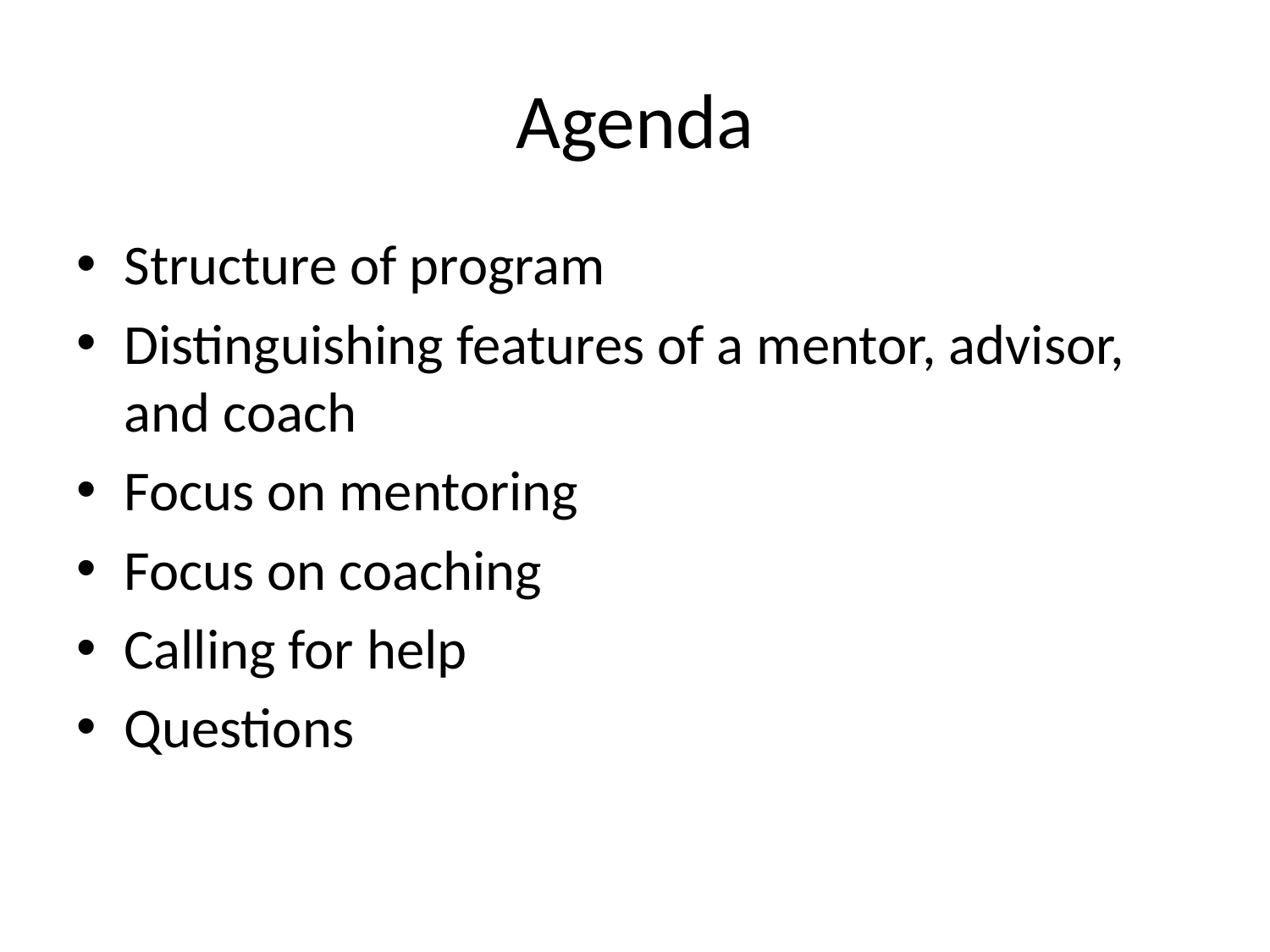

# Agenda
Structure of program
Distinguishing features of a mentor, advisor, and coach
Focus on mentoring
Focus on coaching
Calling for help
Questions

## Slide 4
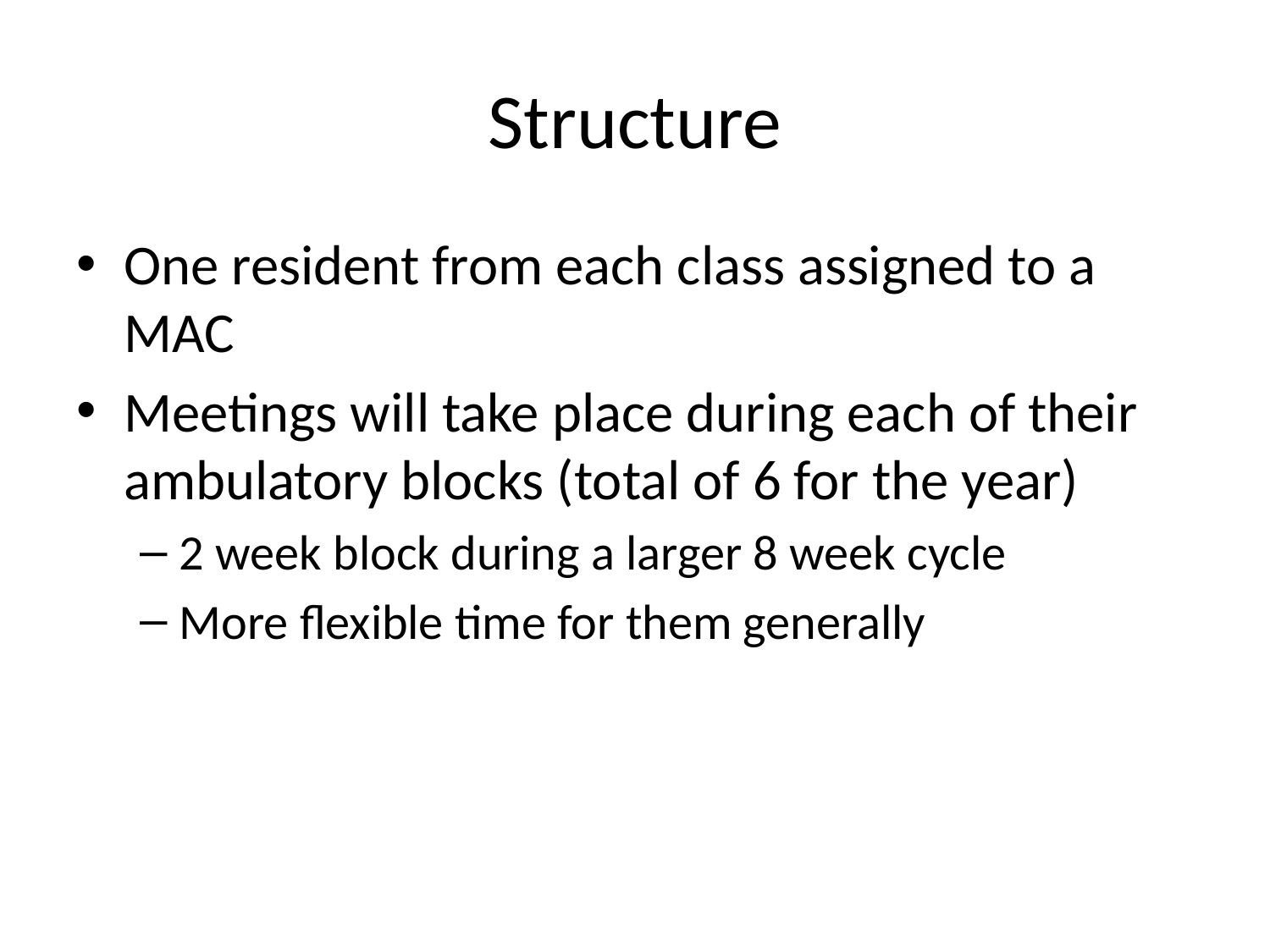

# Structure
One resident from each class assigned to a MAC
Meetings will take place during each of their ambulatory blocks (total of 6 for the year)
2 week block during a larger 8 week cycle
More flexible time for them generally

## Slide 5
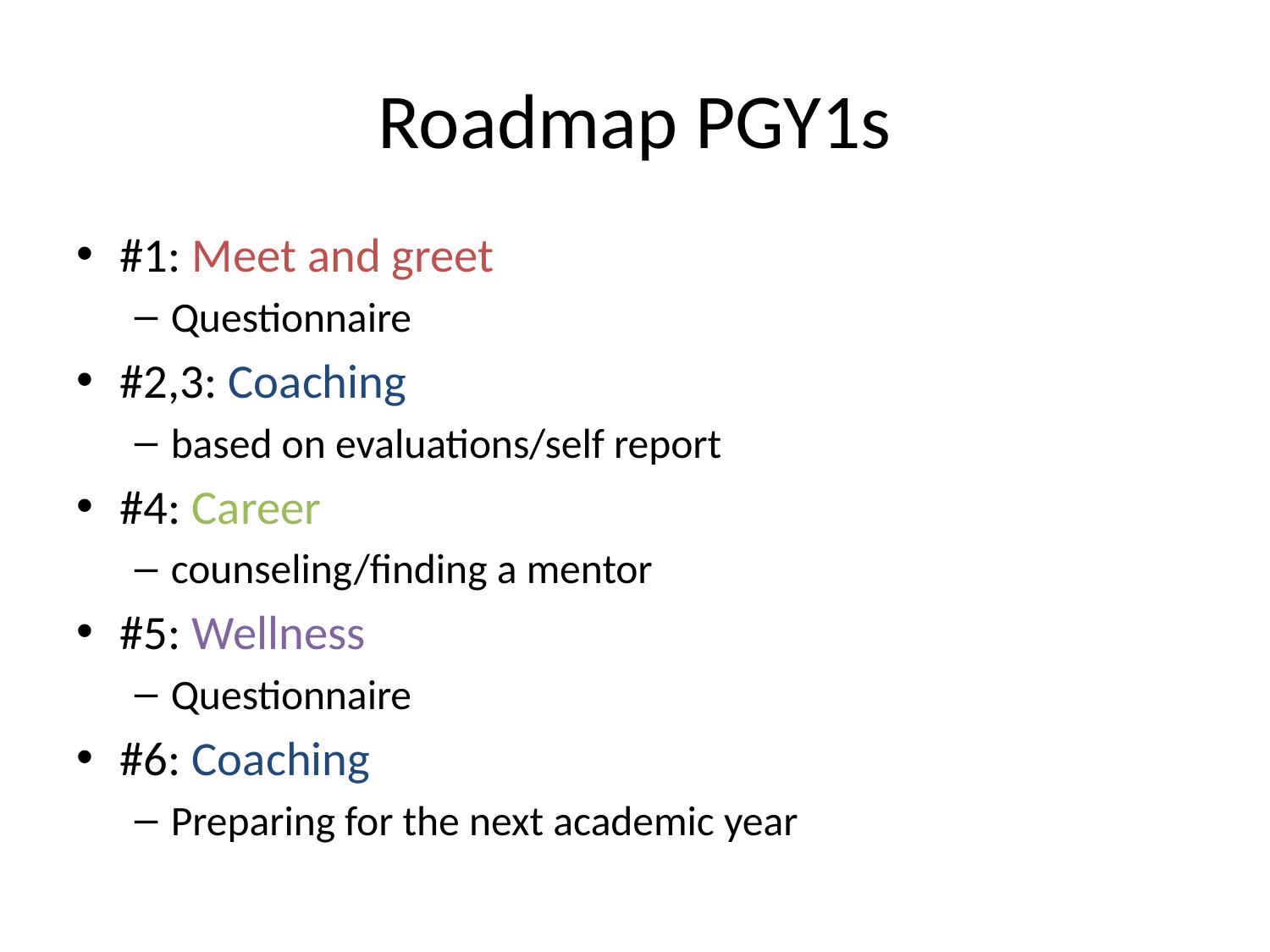

# Roadmap PGY1s
#1: Meet and greet
Questionnaire
#2,3: Coaching
based on evaluations/self report
#4: Career
counseling/finding a mentor
#5: Wellness
Questionnaire
#6: Coaching
Preparing for the next academic year

## Slide 6
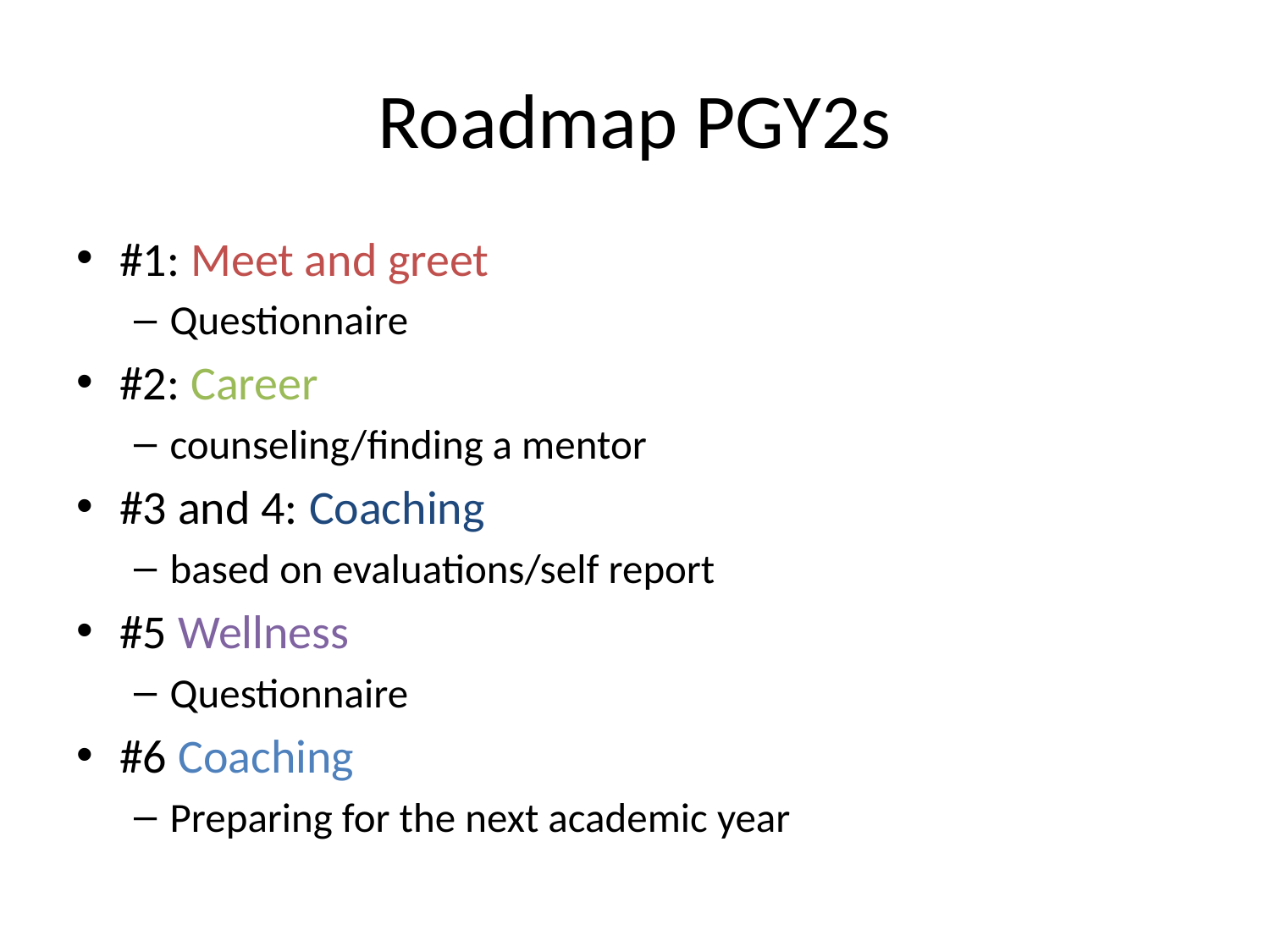

# Roadmap PGY2s
#1: Meet and greet
Questionnaire
#2: Career
counseling/finding a mentor
#3 and 4: Coaching
based on evaluations/self report
#5 Wellness
Questionnaire
#6 Coaching
Preparing for the next academic year

## Slide 7
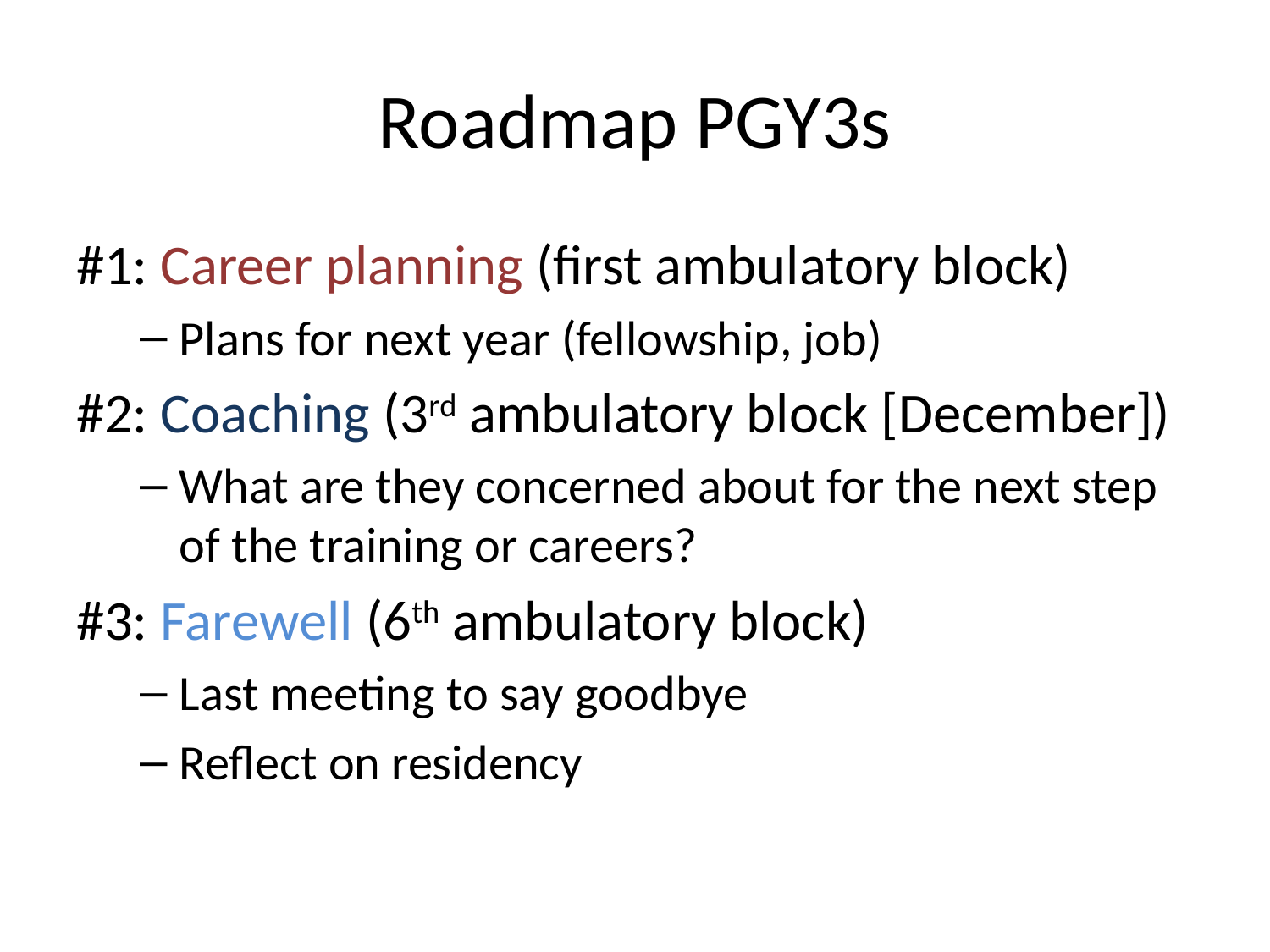

# Roadmap PGY3s
#1: Career planning (first ambulatory block)
Plans for next year (fellowship, job)
#2: Coaching (3rd ambulatory block [December])
What are they concerned about for the next step of the training or careers?
#3: Farewell (6th ambulatory block)
Last meeting to say goodbye
Reflect on residency

## Slide 8
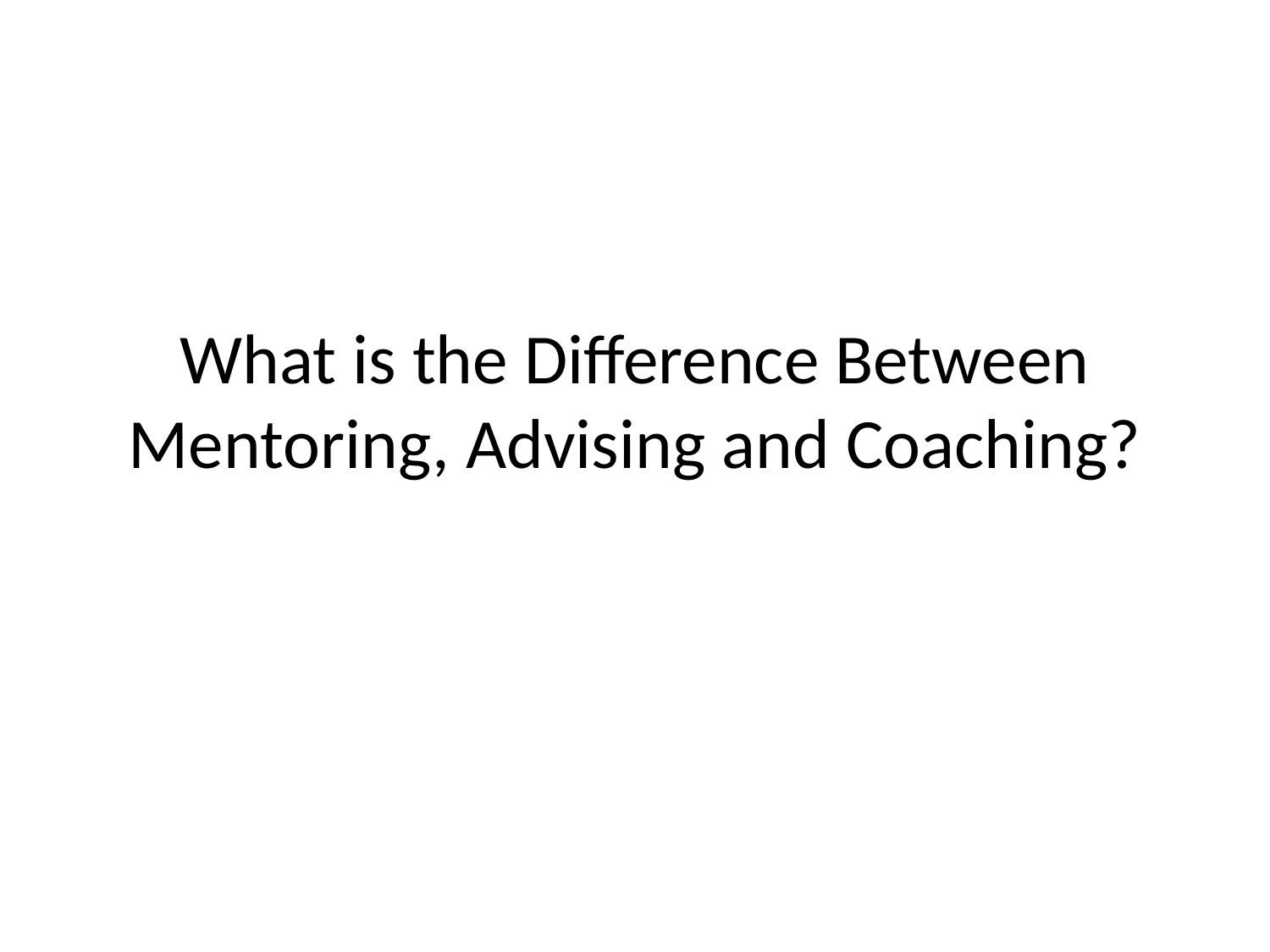

# What is the Difference Between Mentoring, Advising and Coaching?

## Slide 9
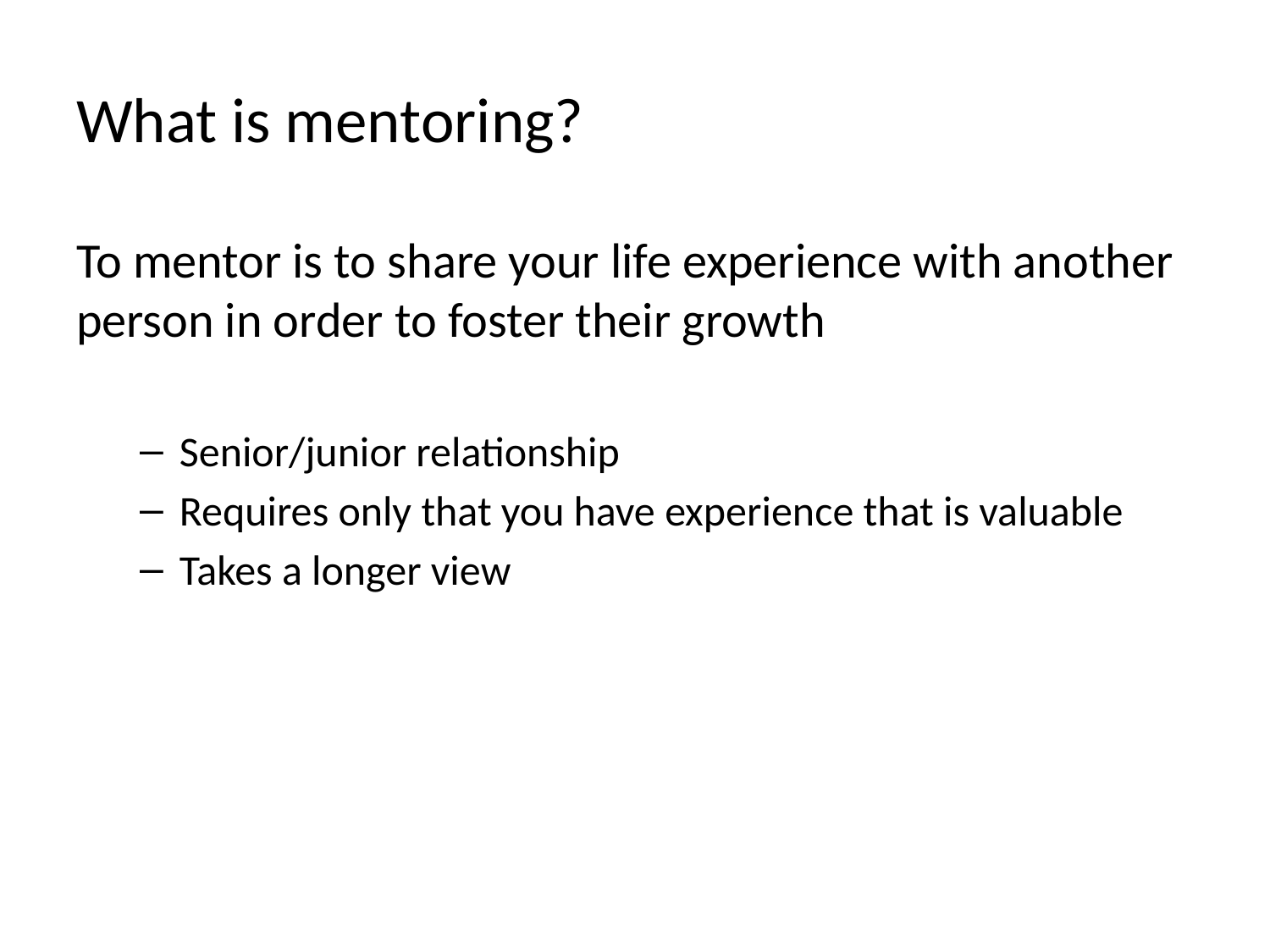

# What is mentoring?
To mentor is to share your life experience with another person in order to foster their growth
Senior/junior relationship
Requires only that you have experience that is valuable
Takes a longer view

## Slide 10
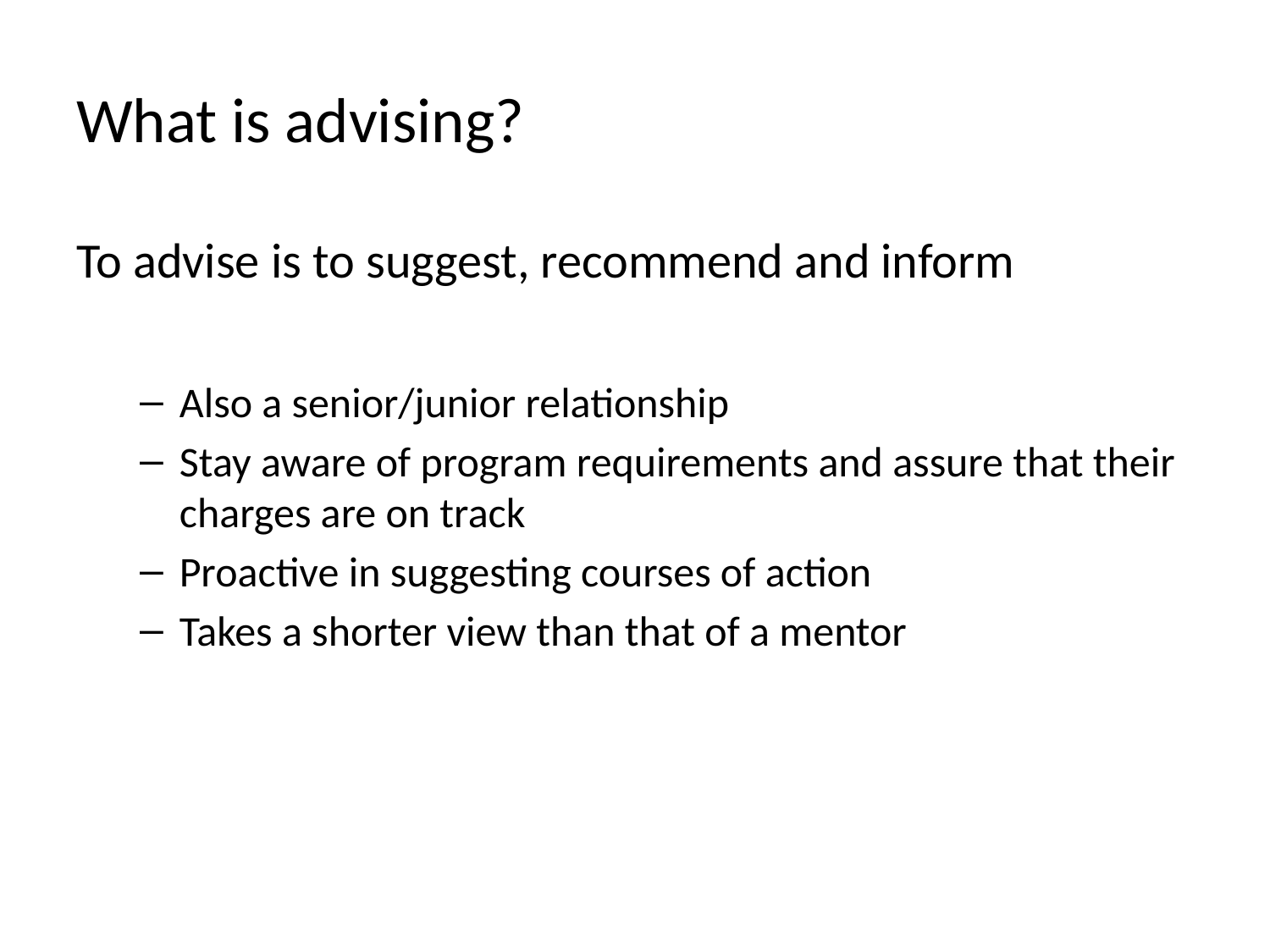

# What is advising?
To advise is to suggest, recommend and inform
Also a senior/junior relationship
Stay aware of program requirements and assure that their charges are on track
Proactive in suggesting courses of action
Takes a shorter view than that of a mentor

## Slide 11
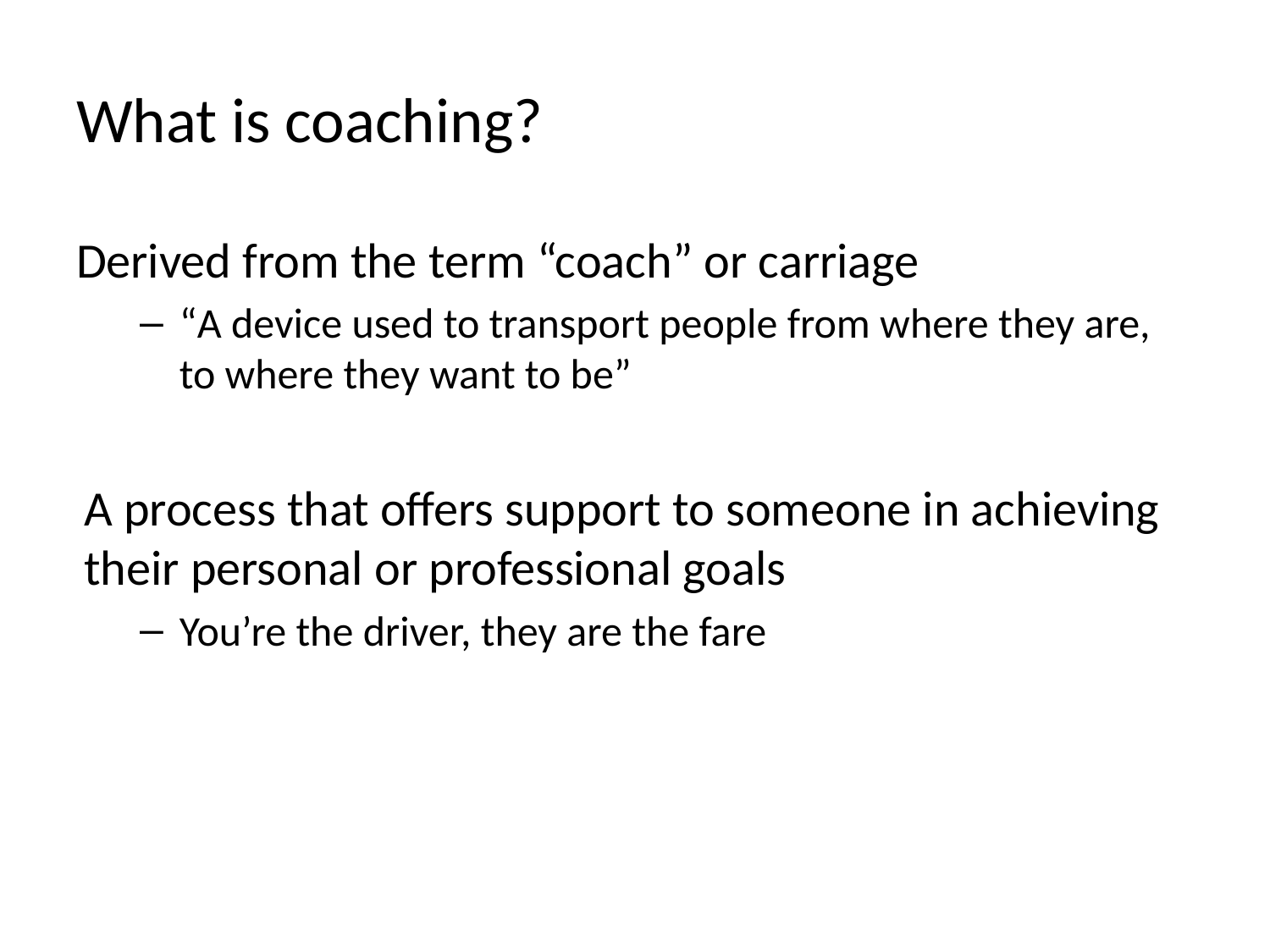

# What is coaching?
Derived from the term “coach” or carriage
“A device used to transport people from where they are, to where they want to be”
A process that offers support to someone in achieving their personal or professional goals
You’re the driver, they are the fare

## Slide 12
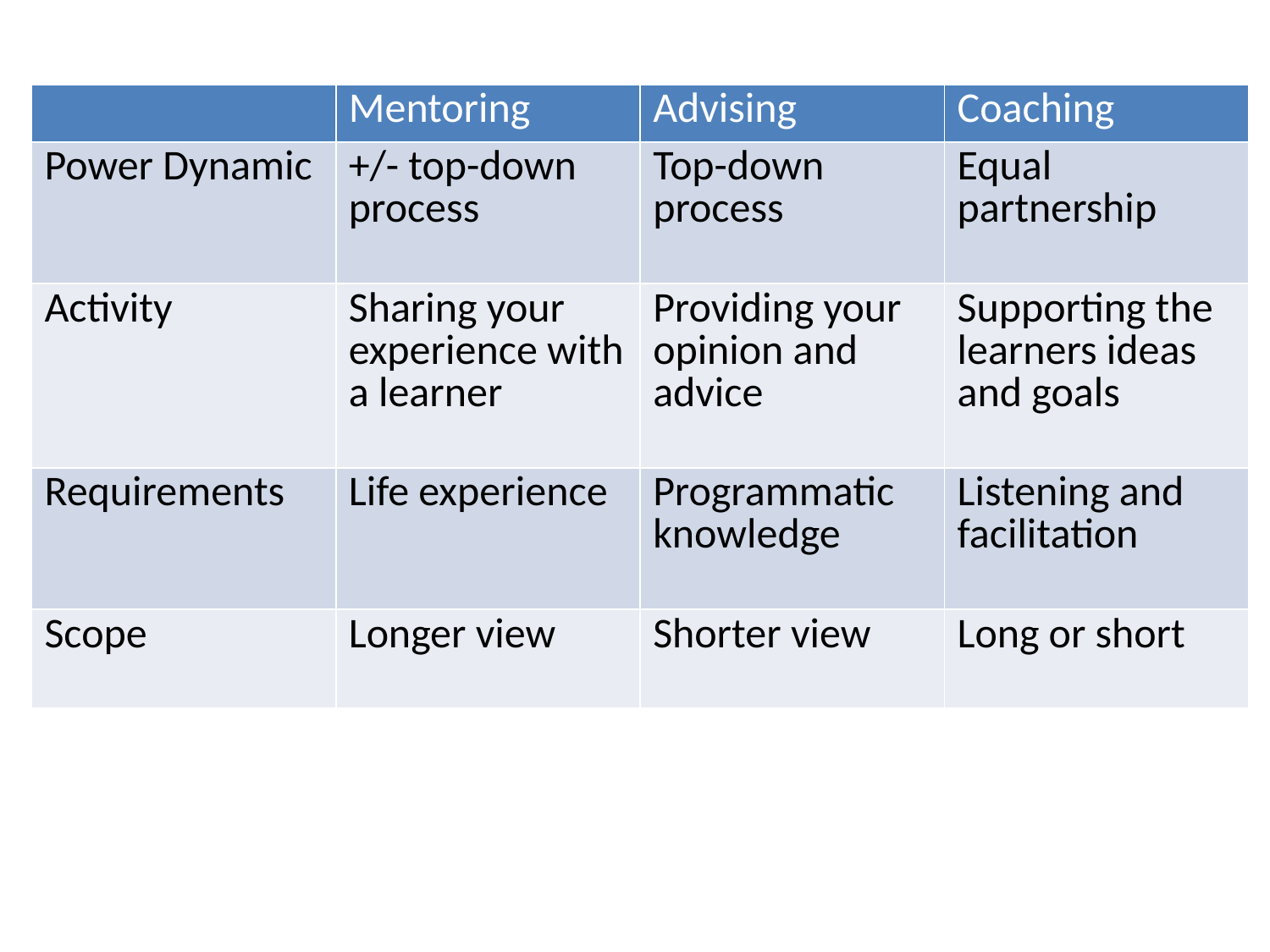

| | Mentoring | Advising | Coaching |
| --- | --- | --- | --- |
| Power Dynamic | +/- top-down process | Top-down process | Equal partnership |
| Activity | Sharing your experience with a learner | Providing your opinion and advice | Supporting the learners ideas and goals |
| Requirements | Life experience | Programmatic knowledge | Listening and facilitation |
| Scope | Longer view | Shorter view | Long or short |

## Slide 13
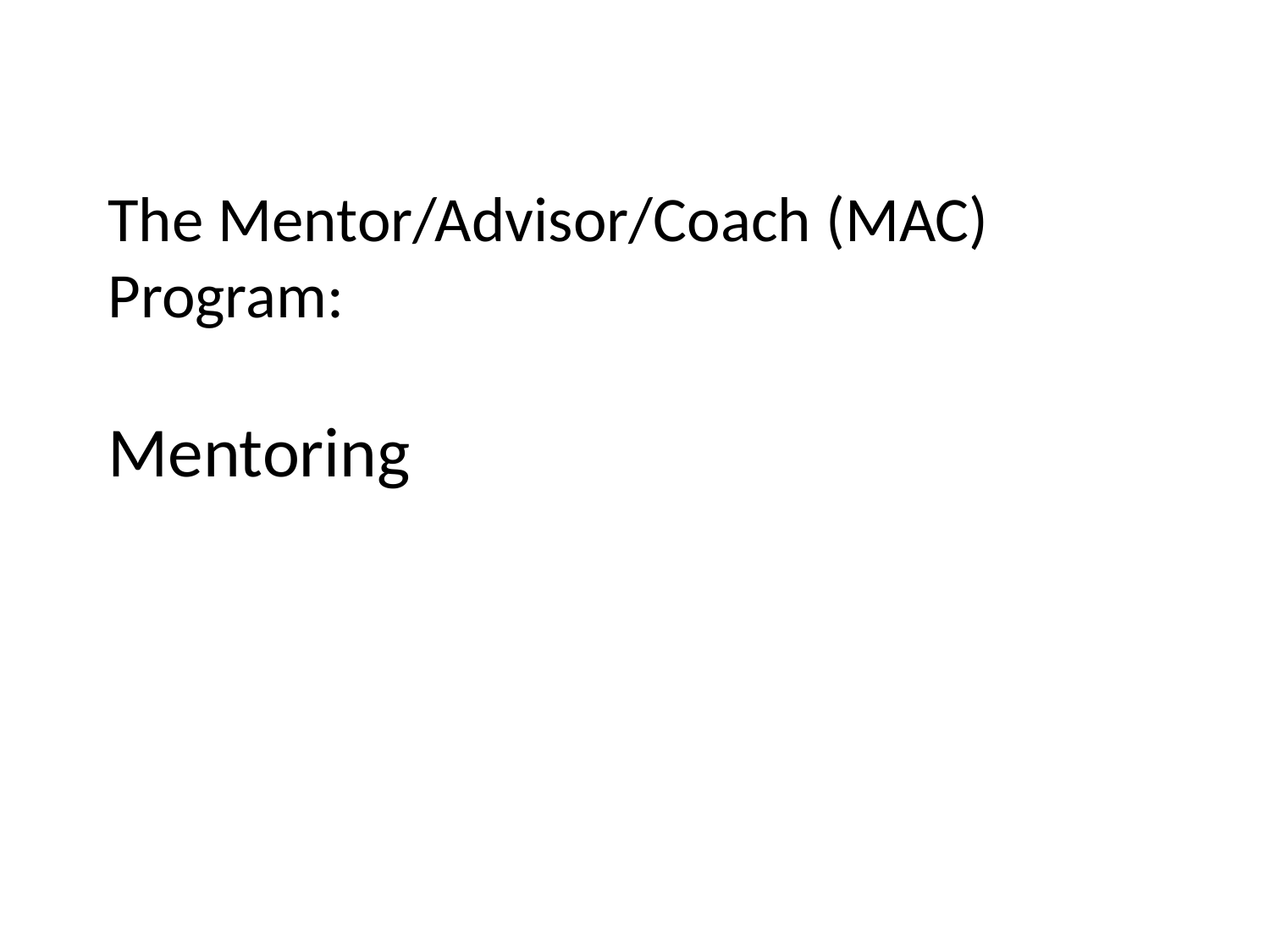

# The Mentor/Advisor/Coach (MAC) Program: Mentoring

## Slide 14
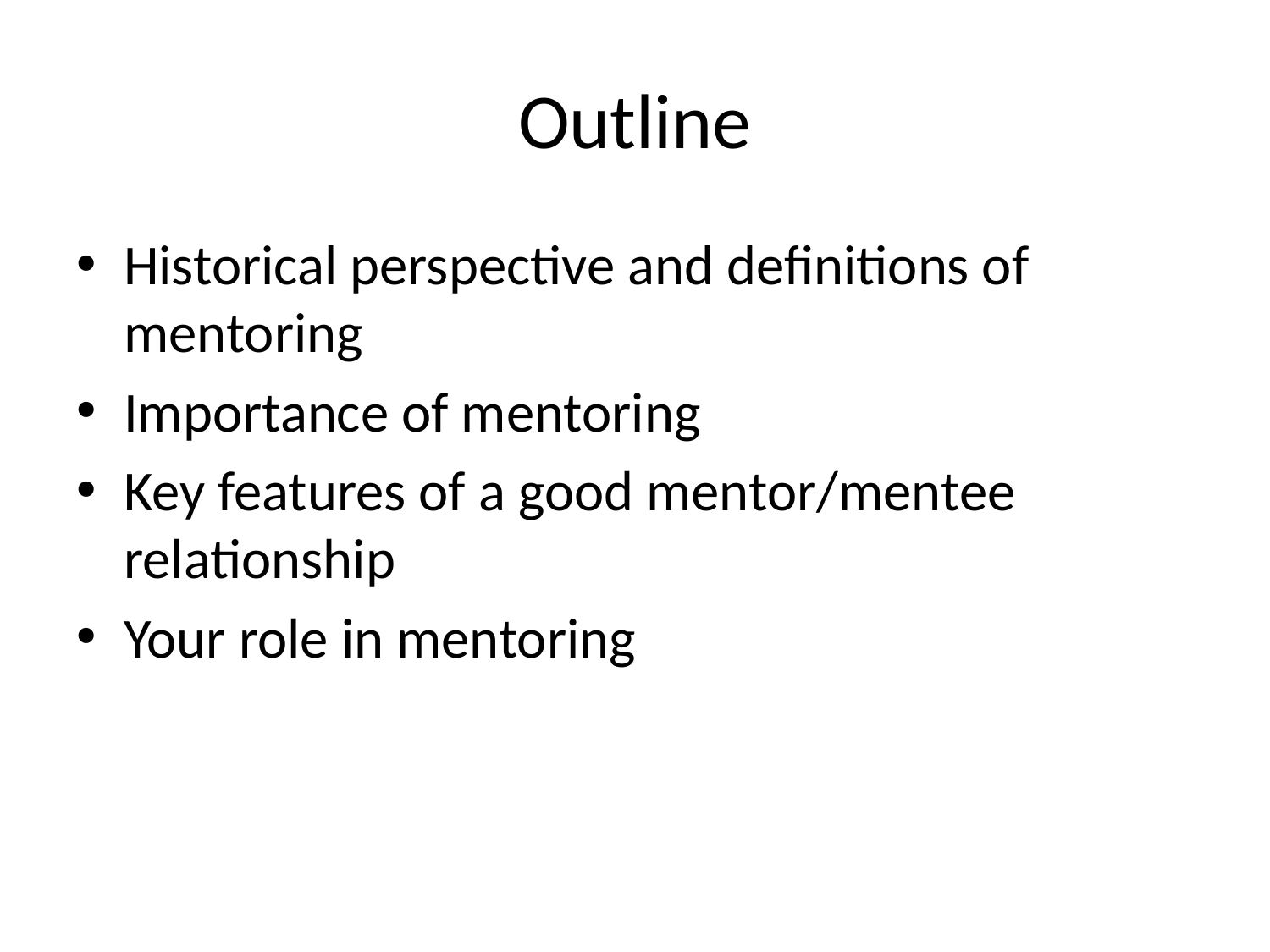

# Outline
Historical perspective and definitions of mentoring
Importance of mentoring
Key features of a good mentor/mentee relationship
Your role in mentoring

## Slide 15
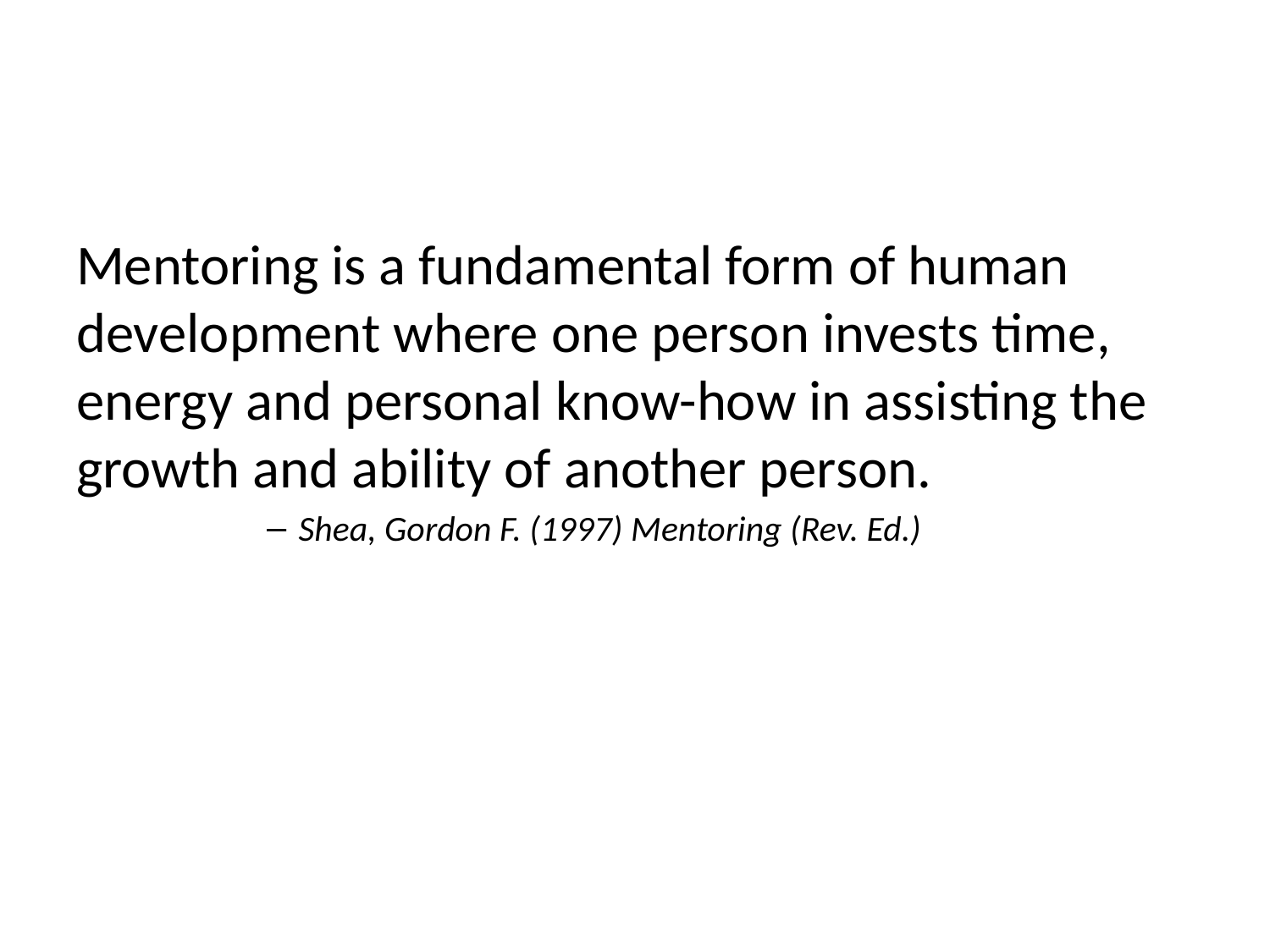

Mentoring is a fundamental form of human development where one person invests time, energy and personal know-how in assisting the growth and ability of another person.
Shea, Gordon F. (1997) Mentoring (Rev. Ed.)

## Slide 16
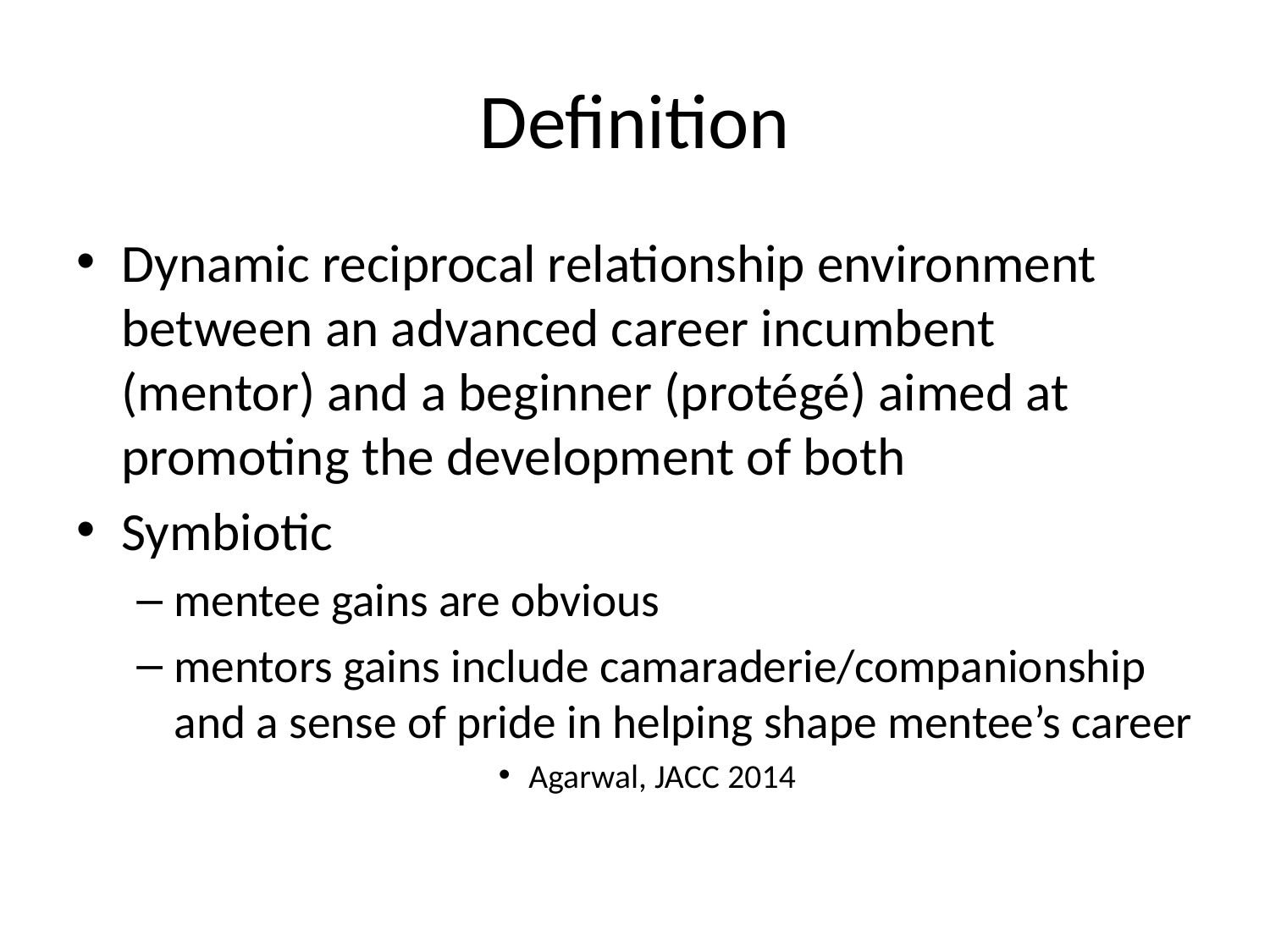

# Definition
Dynamic reciprocal relationship environment between an advanced career incumbent (mentor) and a beginner (protégé) aimed at promoting the development of both
Symbiotic
mentee gains are obvious
mentors gains include camaraderie/companionship and a sense of pride in helping shape mentee’s career
Agarwal, JACC 2014

## Slide 17
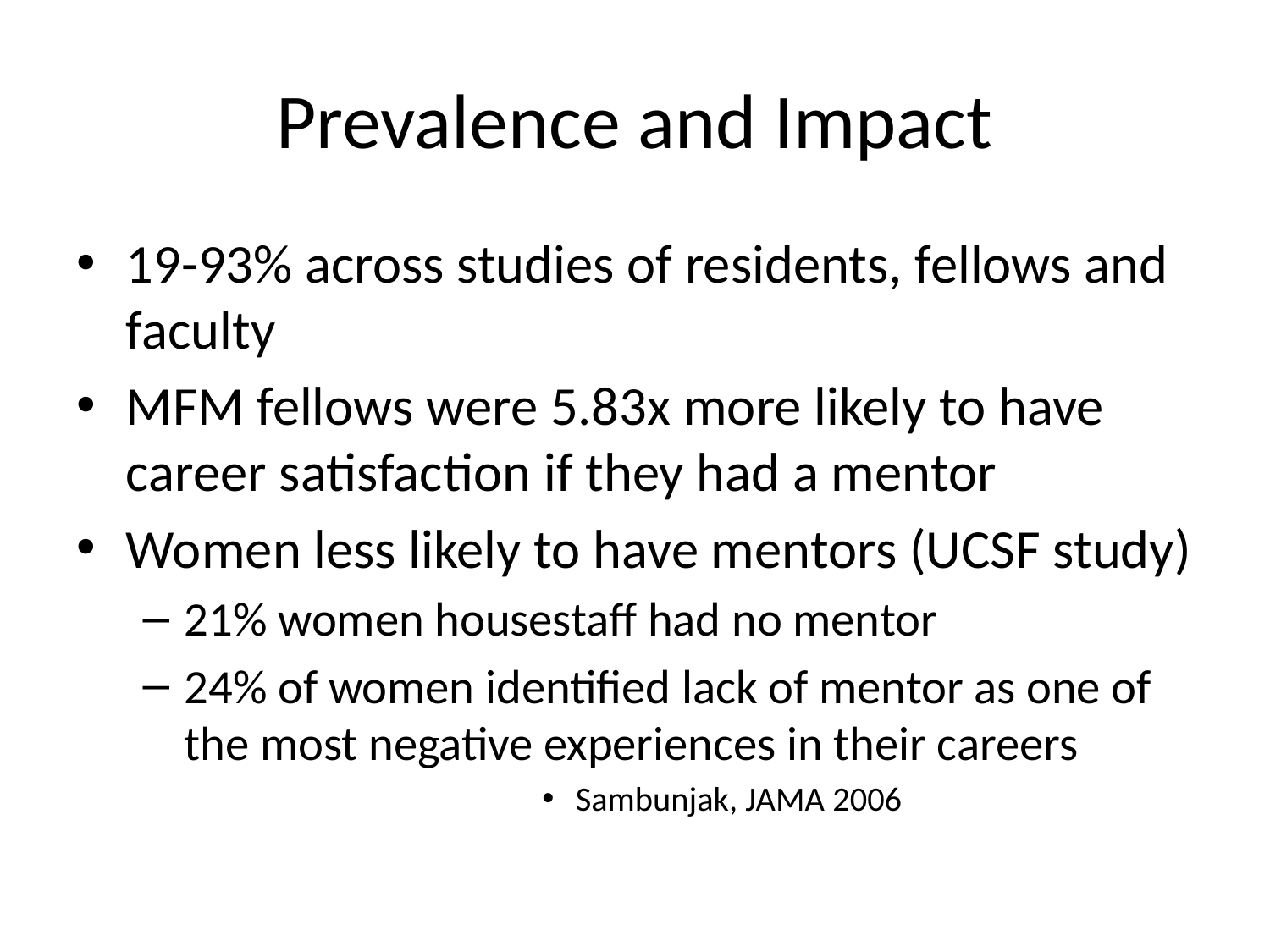

# Prevalence and Impact
19-93% across studies of residents, fellows and faculty
MFM fellows were 5.83x more likely to have career satisfaction if they had a mentor
Women less likely to have mentors (UCSF study)
21% women housestaff had no mentor
24% of women identified lack of mentor as one of the most negative experiences in their careers
Sambunjak, JAMA 2006

## Slide 18
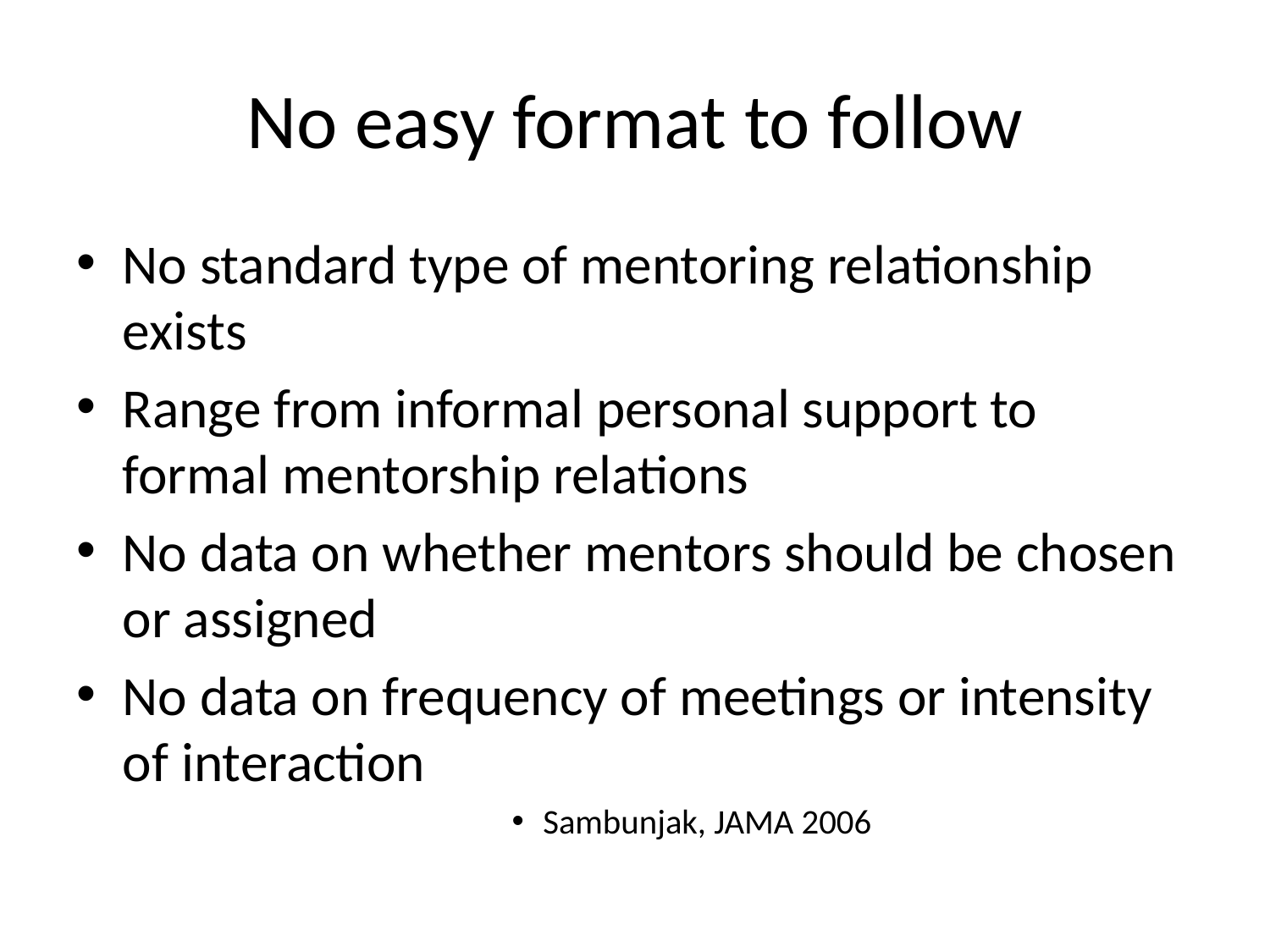

# No easy format to follow
No standard type of mentoring relationship exists
Range from informal personal support to formal mentorship relations
No data on whether mentors should be chosen or assigned
No data on frequency of meetings or intensity of interaction
Sambunjak, JAMA 2006

## Slide 19
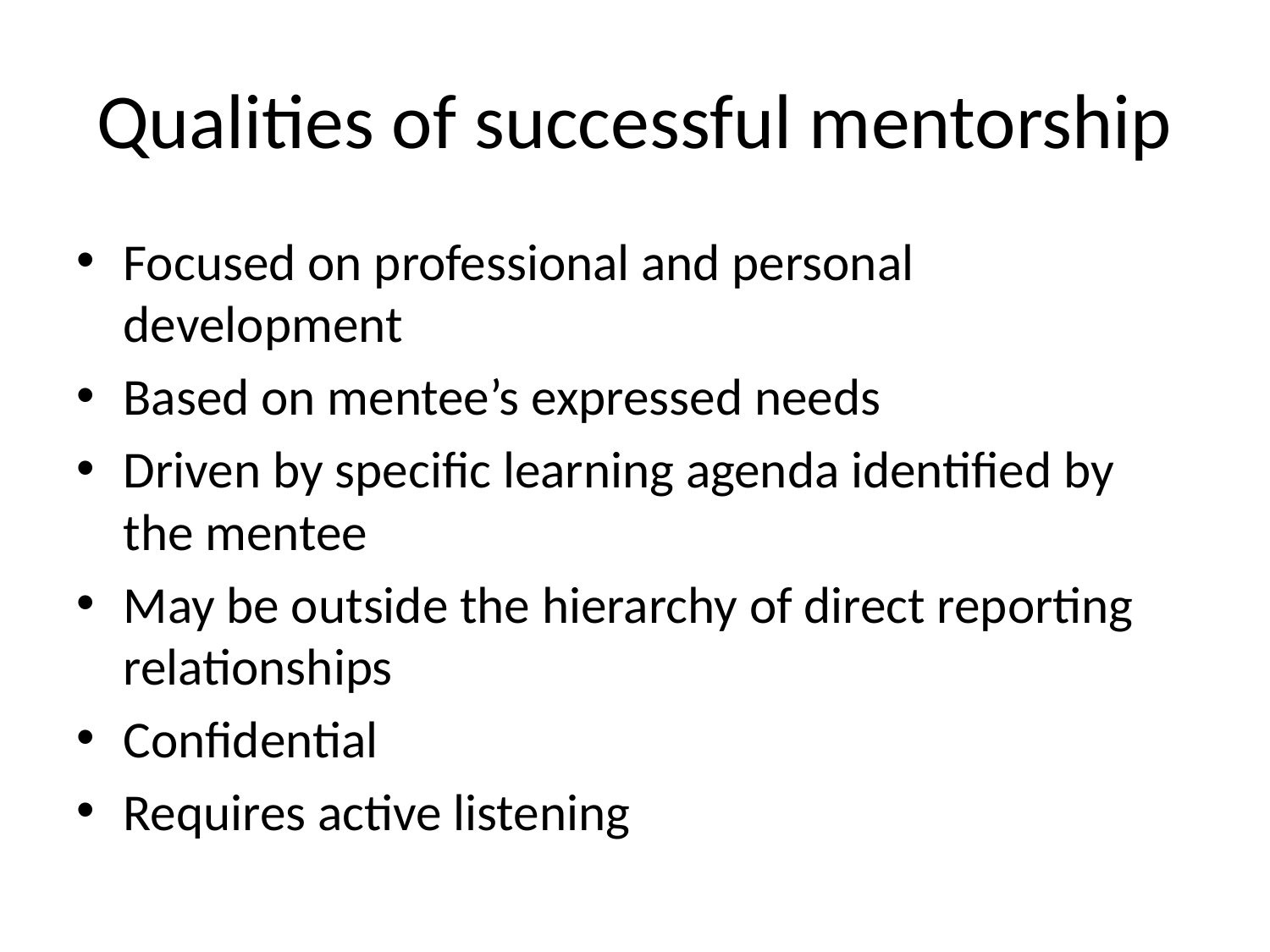

# Qualities of successful mentorship
Focused on professional and personal development
Based on mentee’s expressed needs
Driven by specific learning agenda identified by the mentee
May be outside the hierarchy of direct reporting relationships
Confidential
Requires active listening

## Slide 20
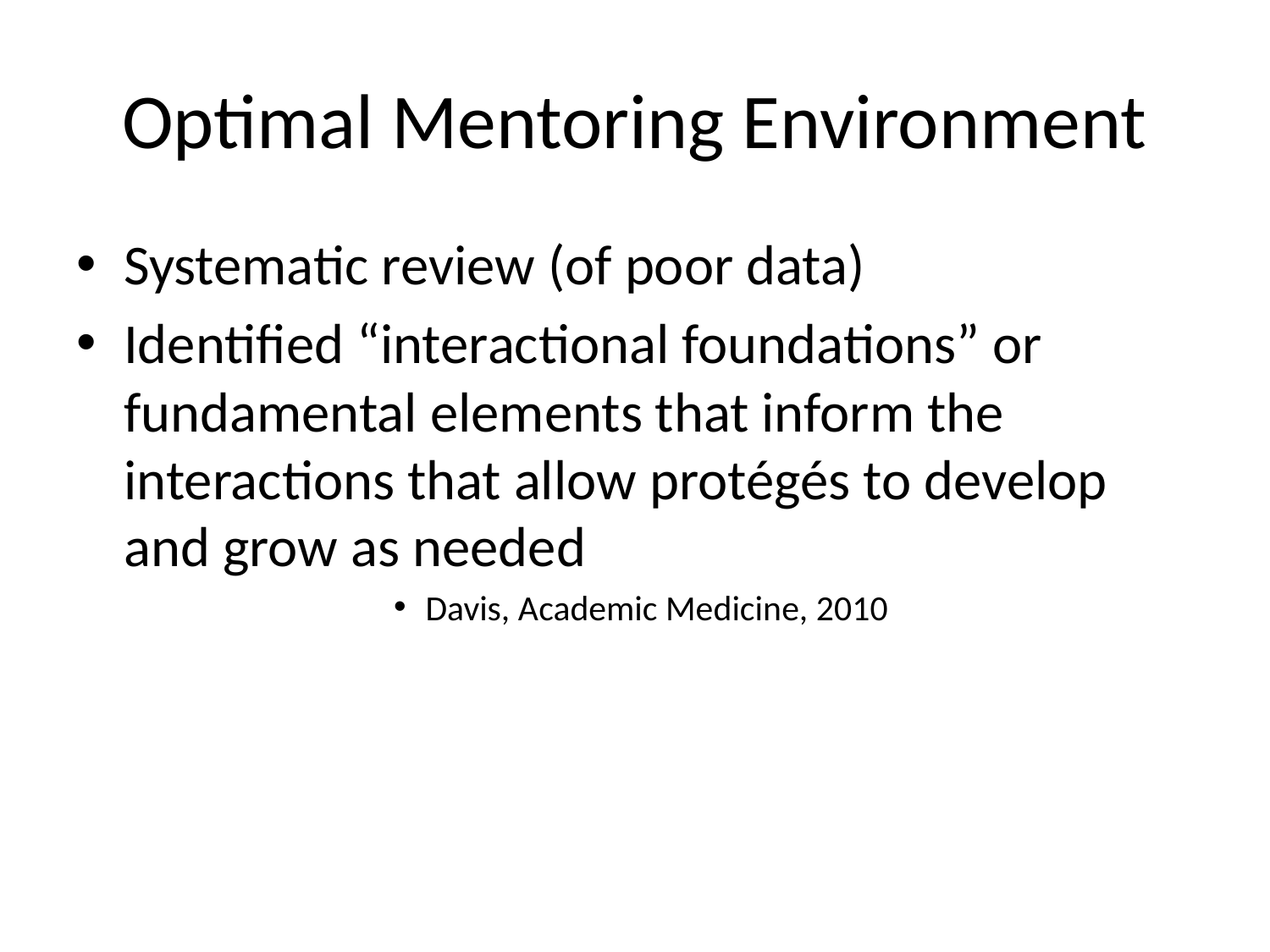

# Optimal Mentoring Environment
Systematic review (of poor data)
Identified “interactional foundations” or fundamental elements that inform the interactions that allow protégés to develop and grow as needed
Davis, Academic Medicine, 2010

## Slide 21
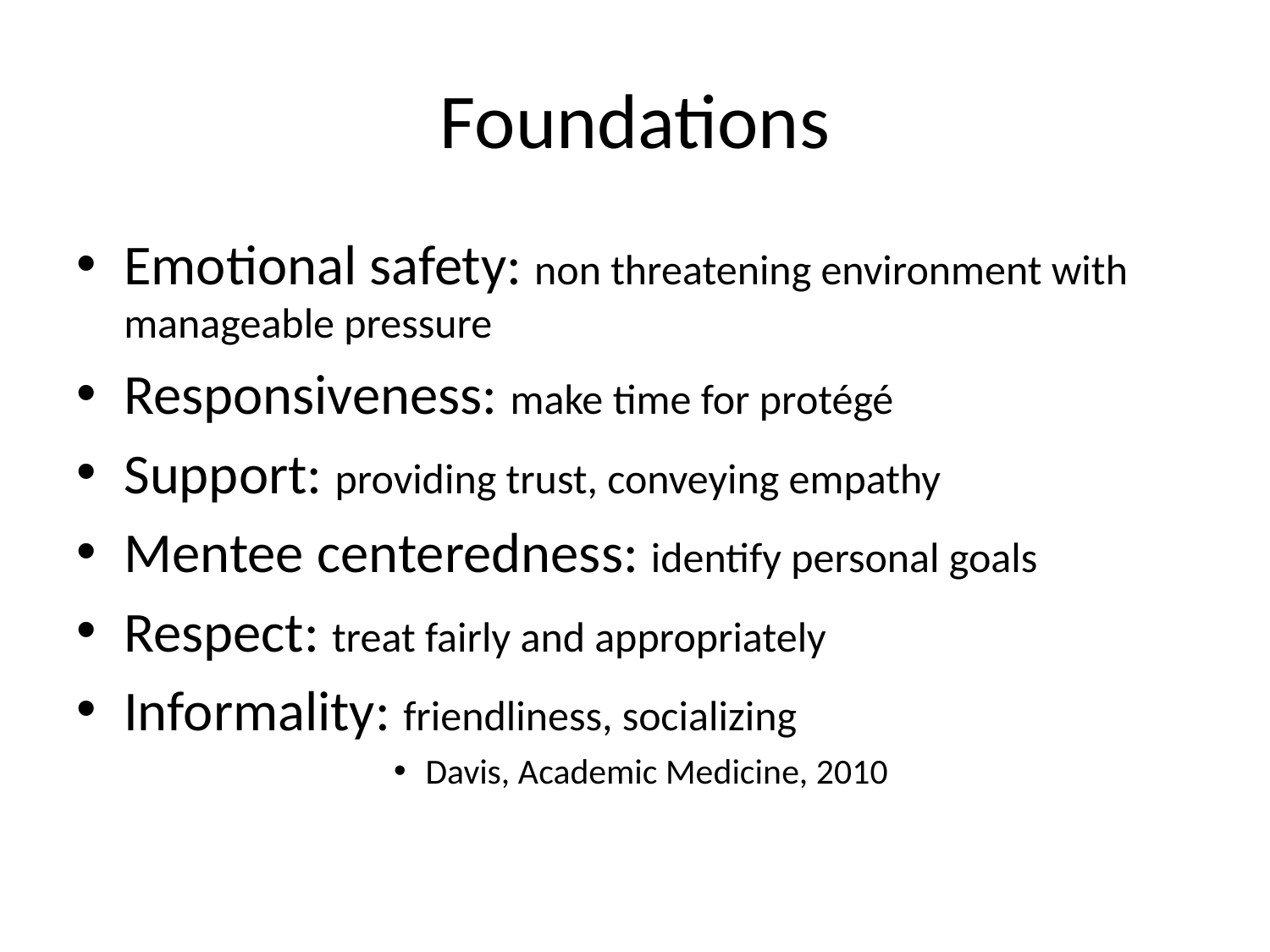

# Foundations
Emotional safety: non threatening environment with manageable pressure
Responsiveness: make time for protégé
Support: providing trust, conveying empathy
Mentee centeredness: identify personal goals
Respect: treat fairly and appropriately
Informality: friendliness, socializing
Davis, Academic Medicine, 2010

## Slide 22
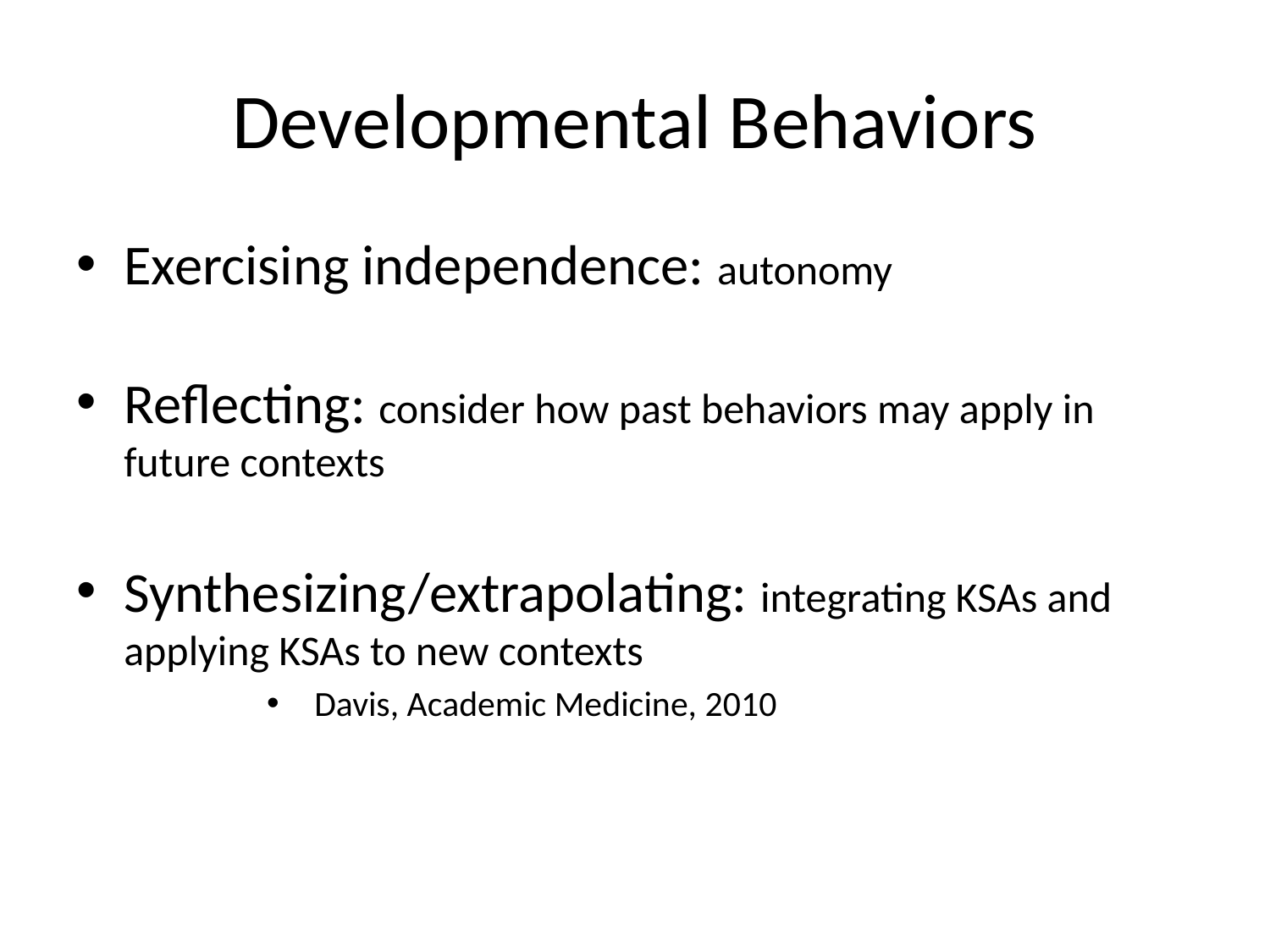

# Developmental Behaviors
Exercising independence: autonomy
Reflecting: consider how past behaviors may apply in future contexts
Synthesizing/extrapolating: integrating KSAs and applying KSAs to new contexts
Davis, Academic Medicine, 2010

## Slide 23
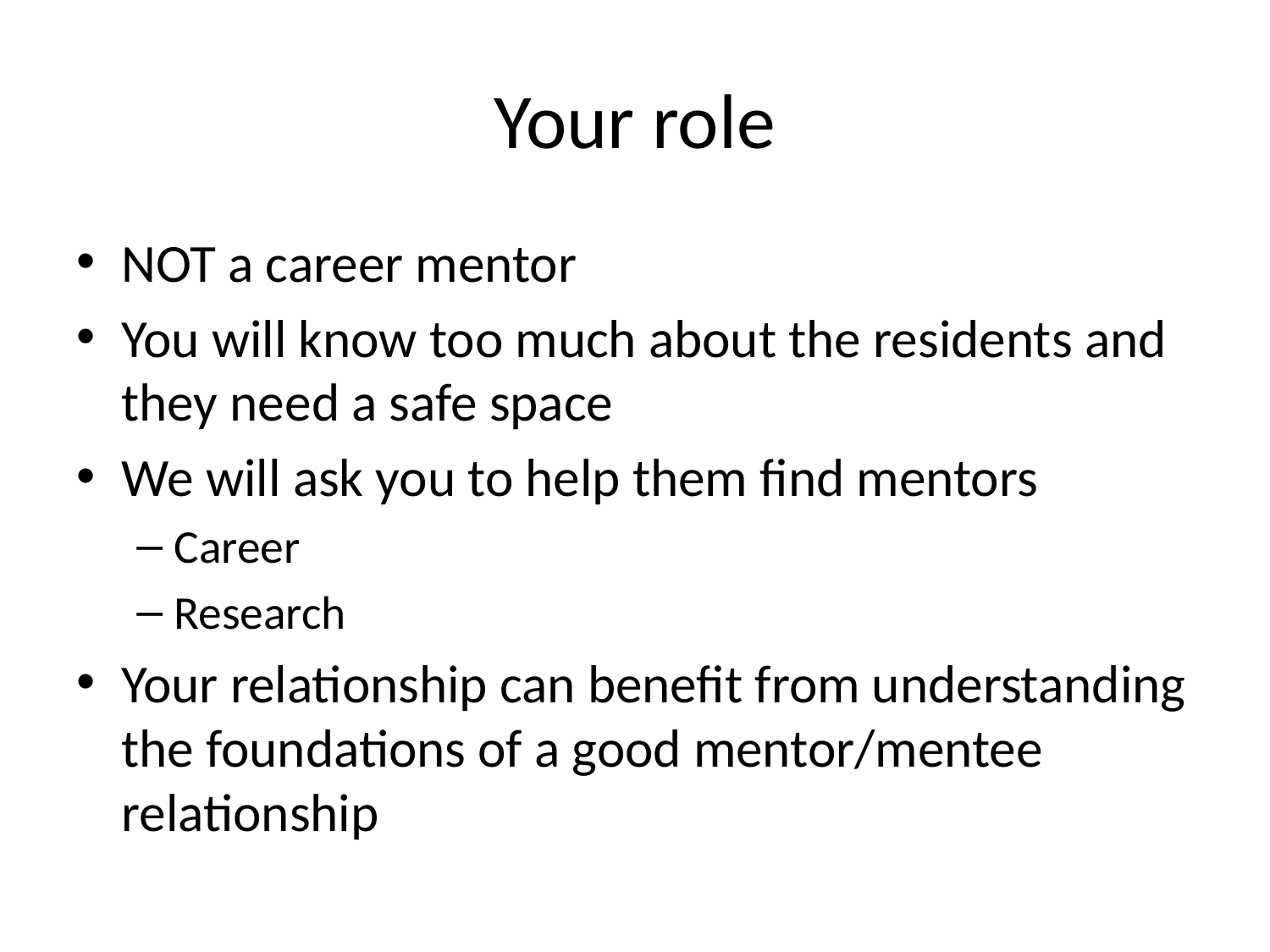

# Your role
NOT a career mentor
You will know too much about the residents and they need a safe space
We will ask you to help them find mentors
Career
Research
Your relationship can benefit from understanding the foundations of a good mentor/mentee relationship

## Slide 24
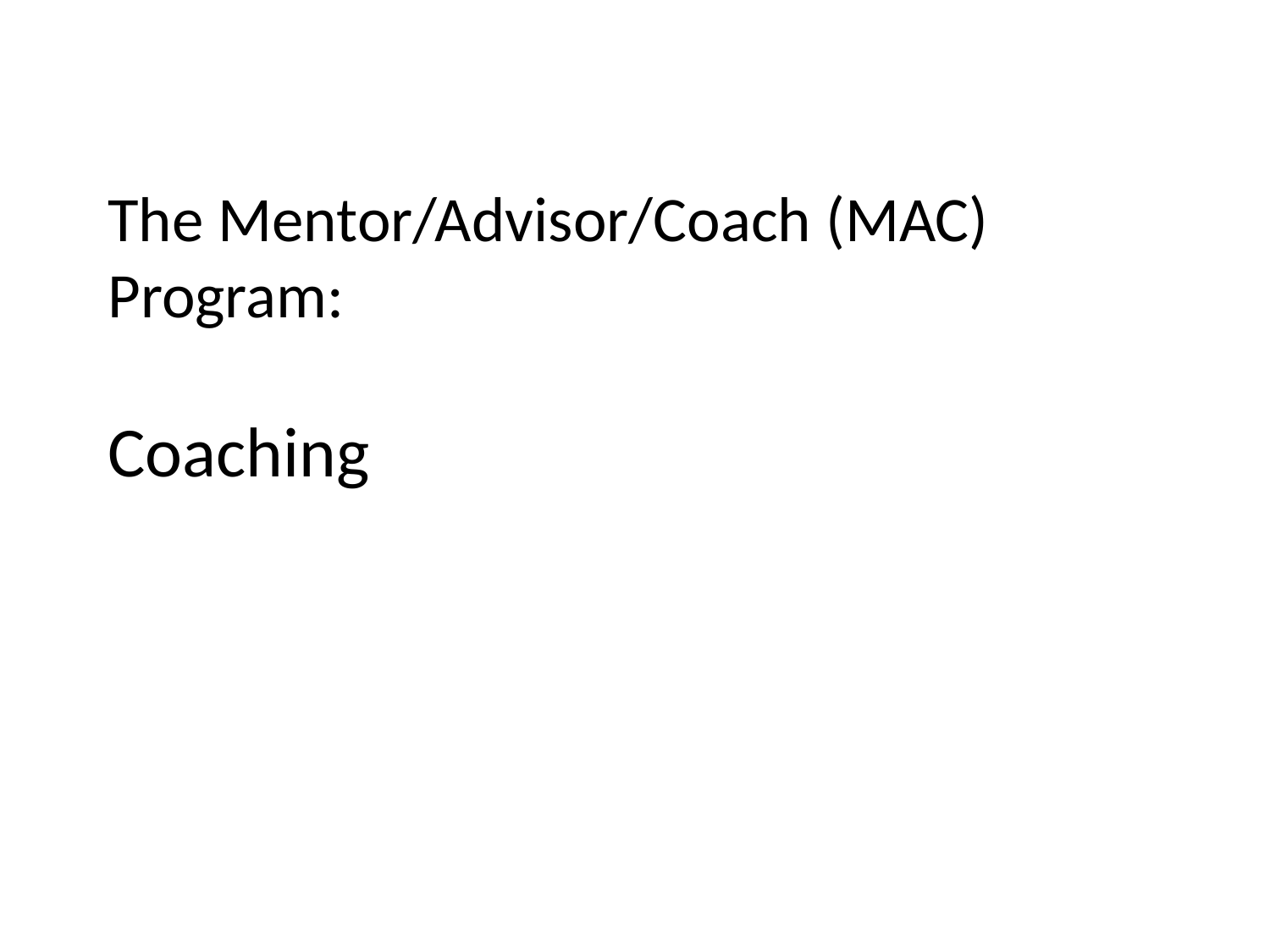

# The Mentor/Advisor/Coach (MAC) Program: Coaching

## Slide 25
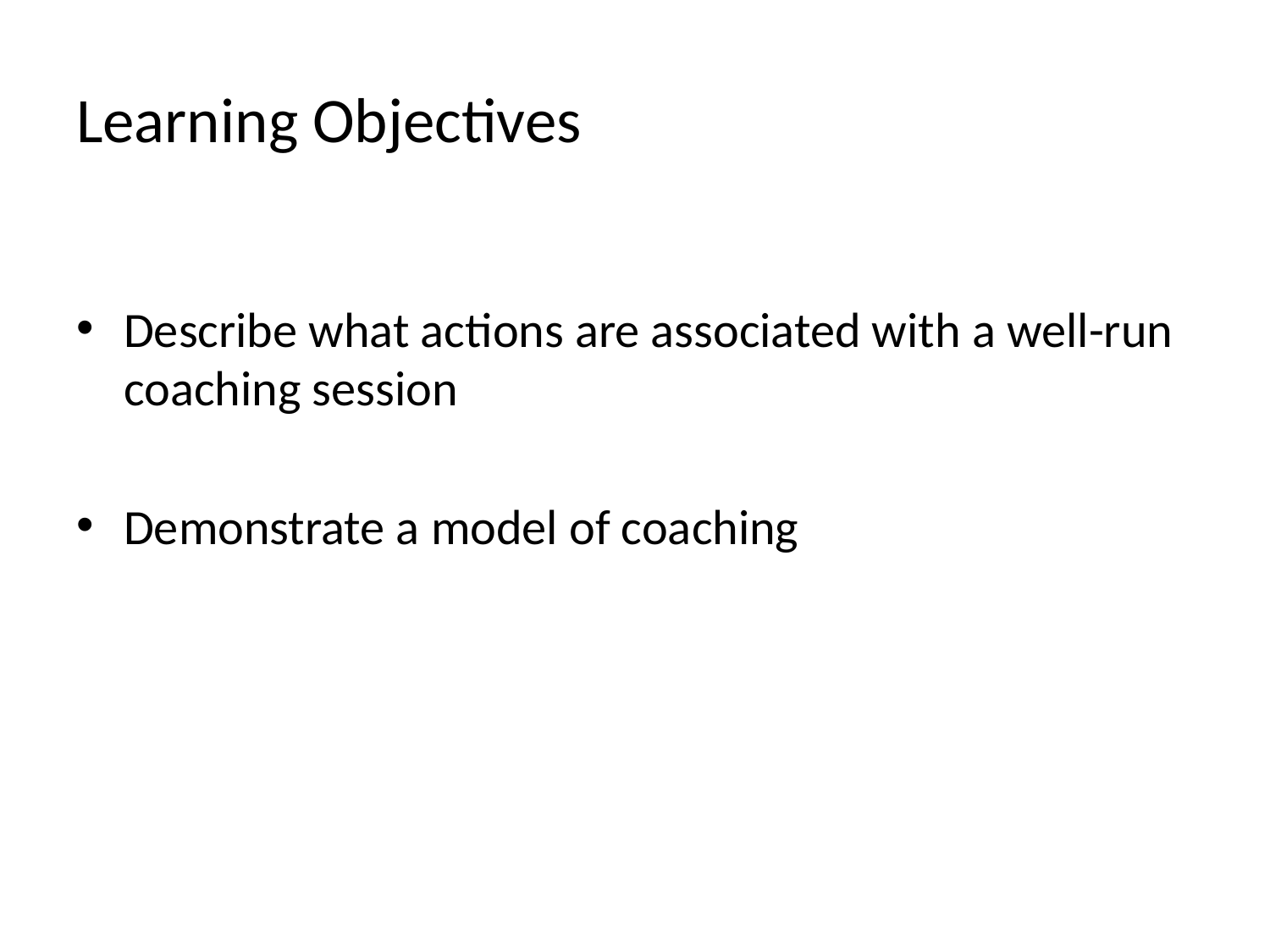

# Learning Objectives
Describe what actions are associated with a well-run coaching session
Demonstrate a model of coaching

## Slide 26
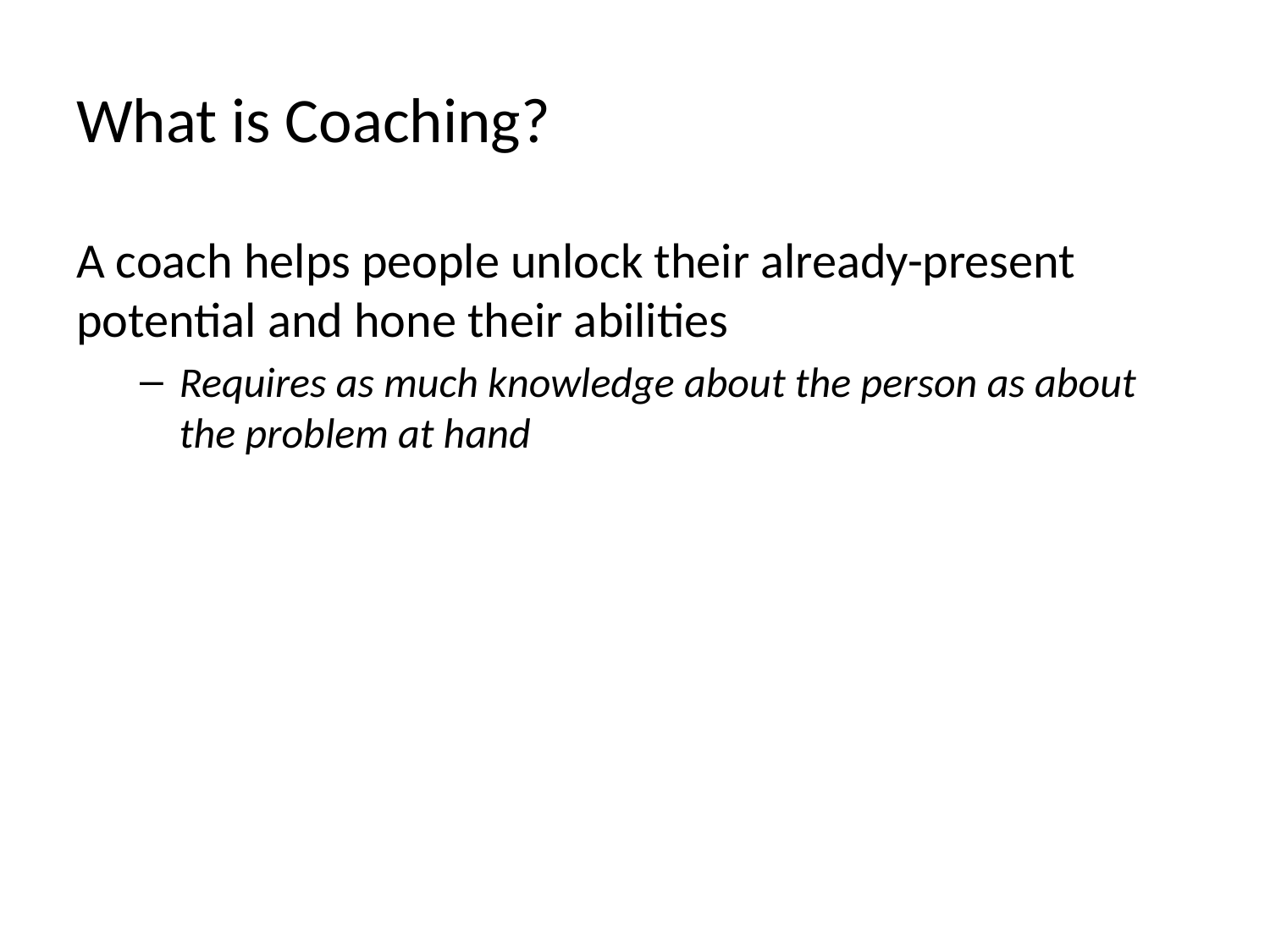

# What is Coaching?
A coach helps people unlock their already-present potential and hone their abilities
Requires as much knowledge about the person as about the problem at hand

## Slide 27
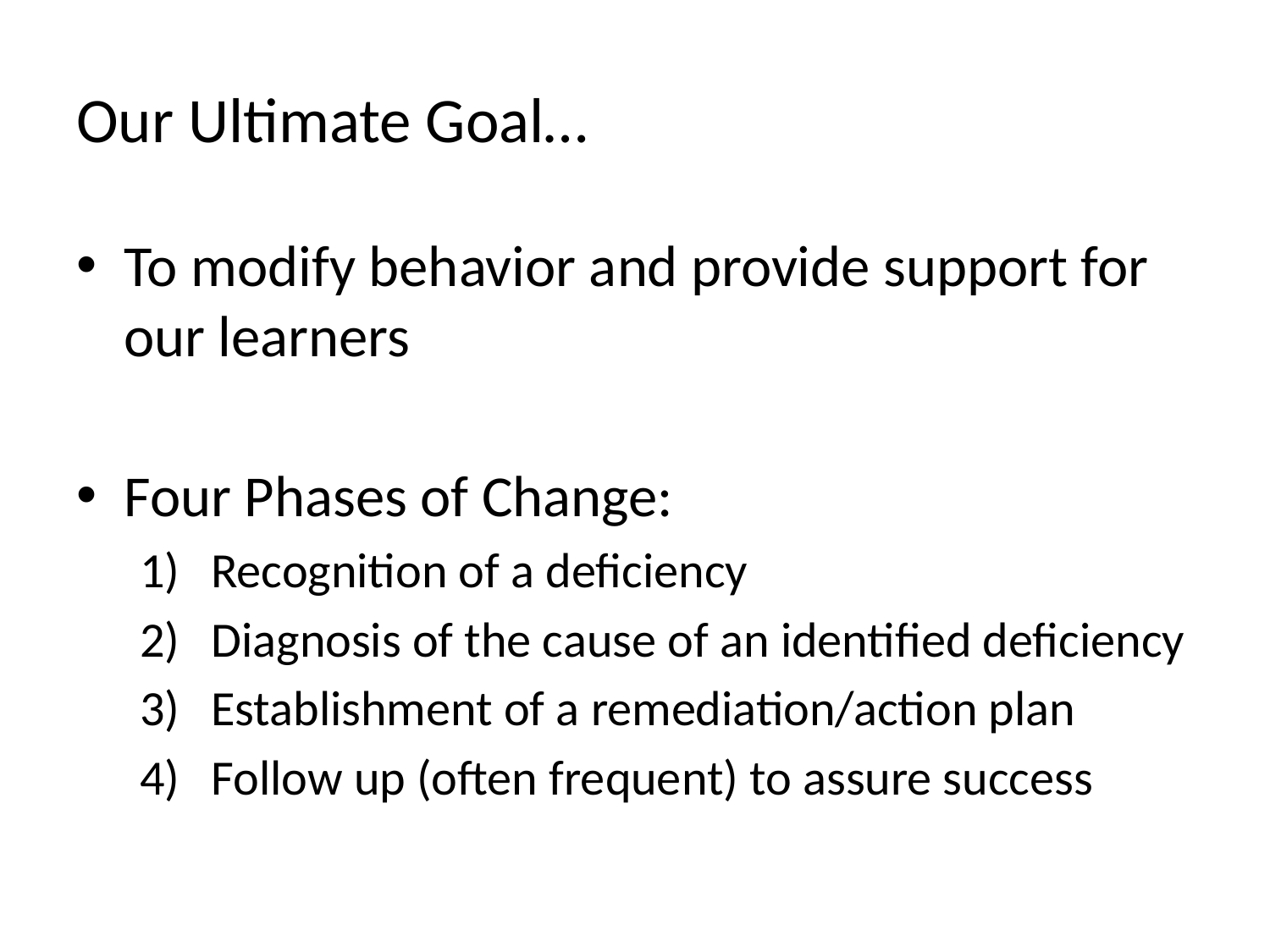

# Our Ultimate Goal…
To modify behavior and provide support for our learners
Four Phases of Change:
Recognition of a deficiency
Diagnosis of the cause of an identified deficiency
Establishment of a remediation/action plan
Follow up (often frequent) to assure success

## Slide 28
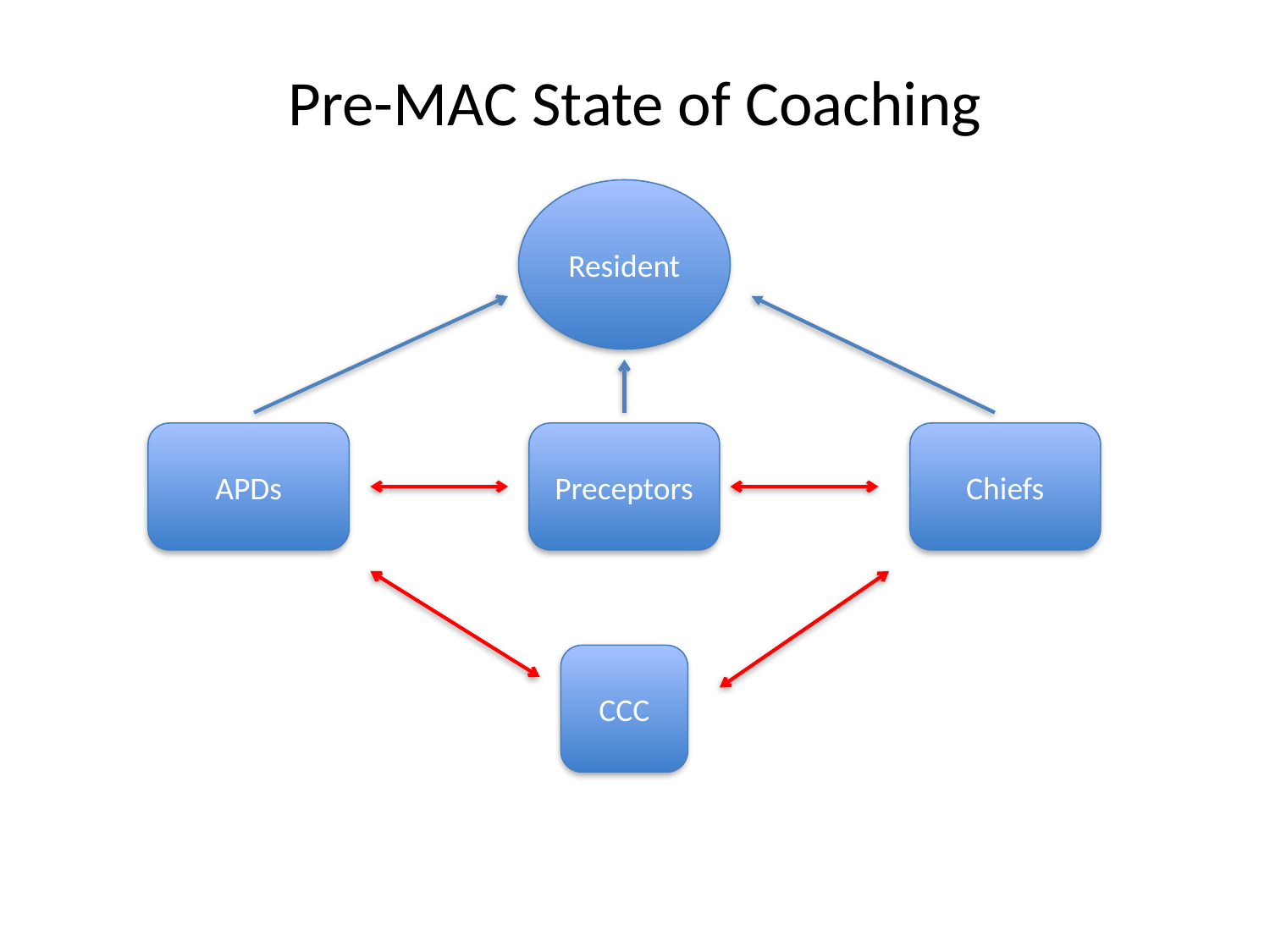

# Pre-MAC State of Coaching
Resident
APDs
Preceptors
Chiefs
CCC

## Slide 29
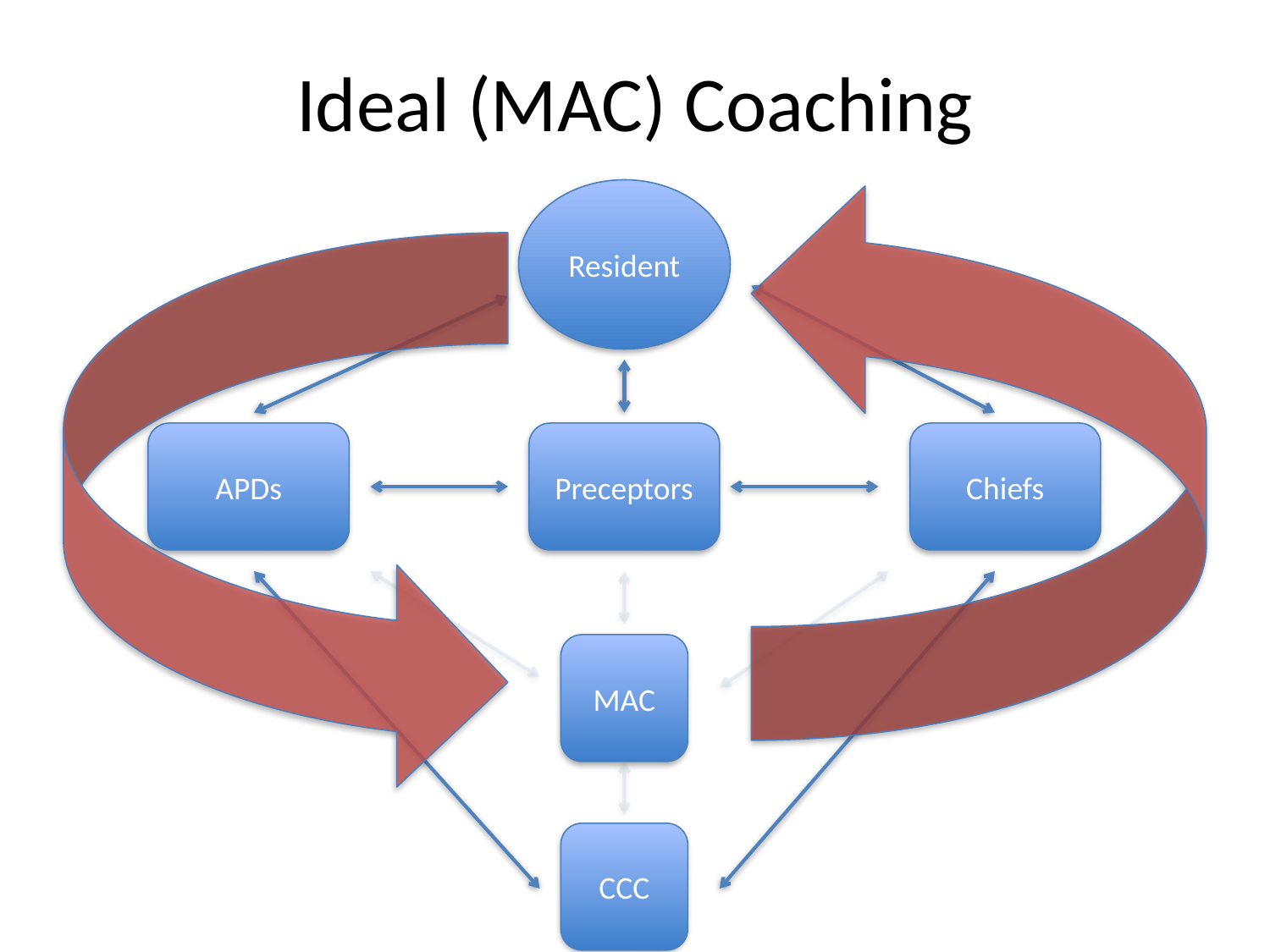

# Ideal (MAC) Coaching
Resident
APDs
Preceptors
Chiefs
MAC
CCC

## Slide 30
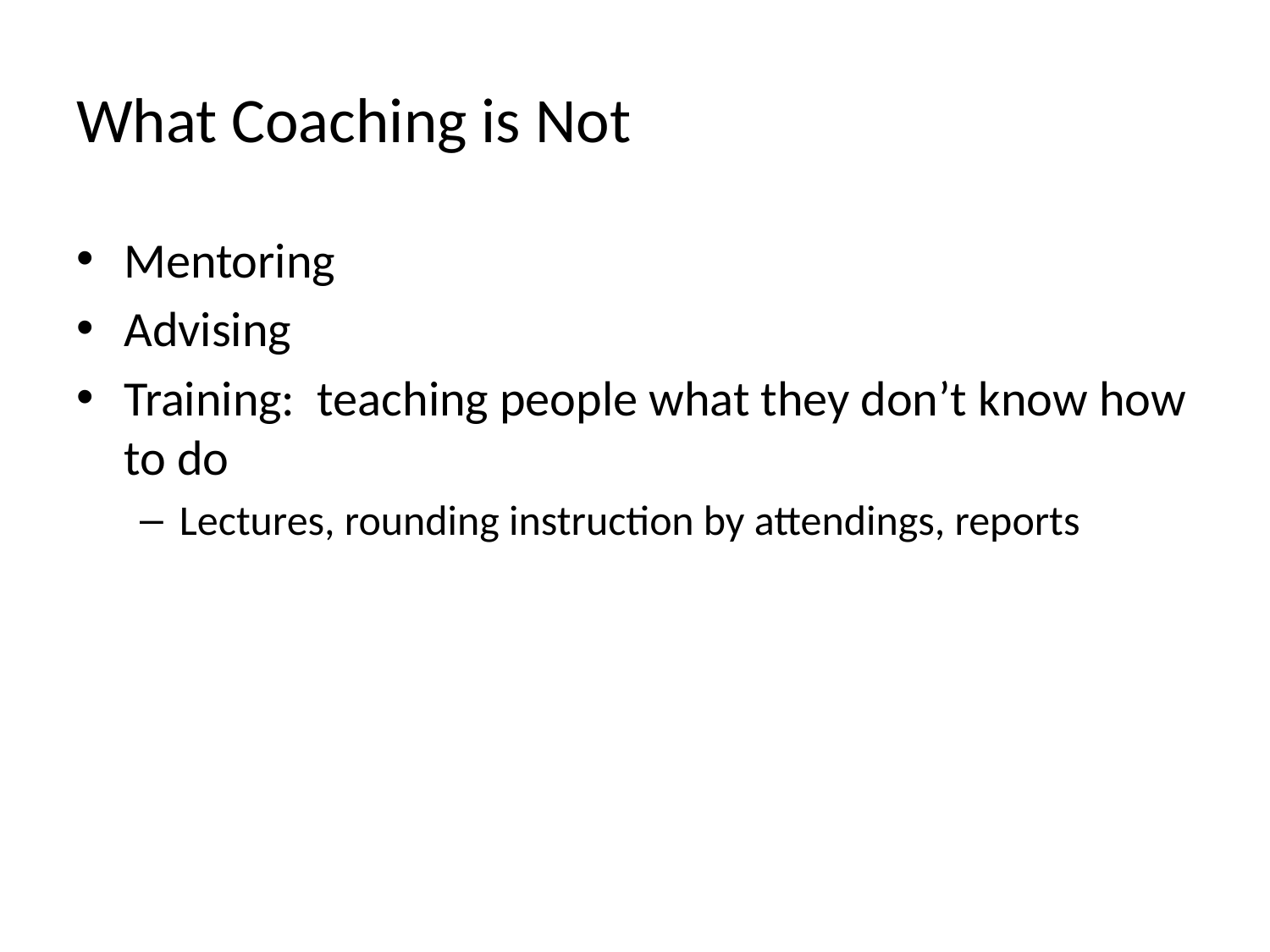

# What Coaching is Not
Mentoring
Advising
Training: teaching people what they don’t know how to do
Lectures, rounding instruction by attendings, reports

## Slide 31
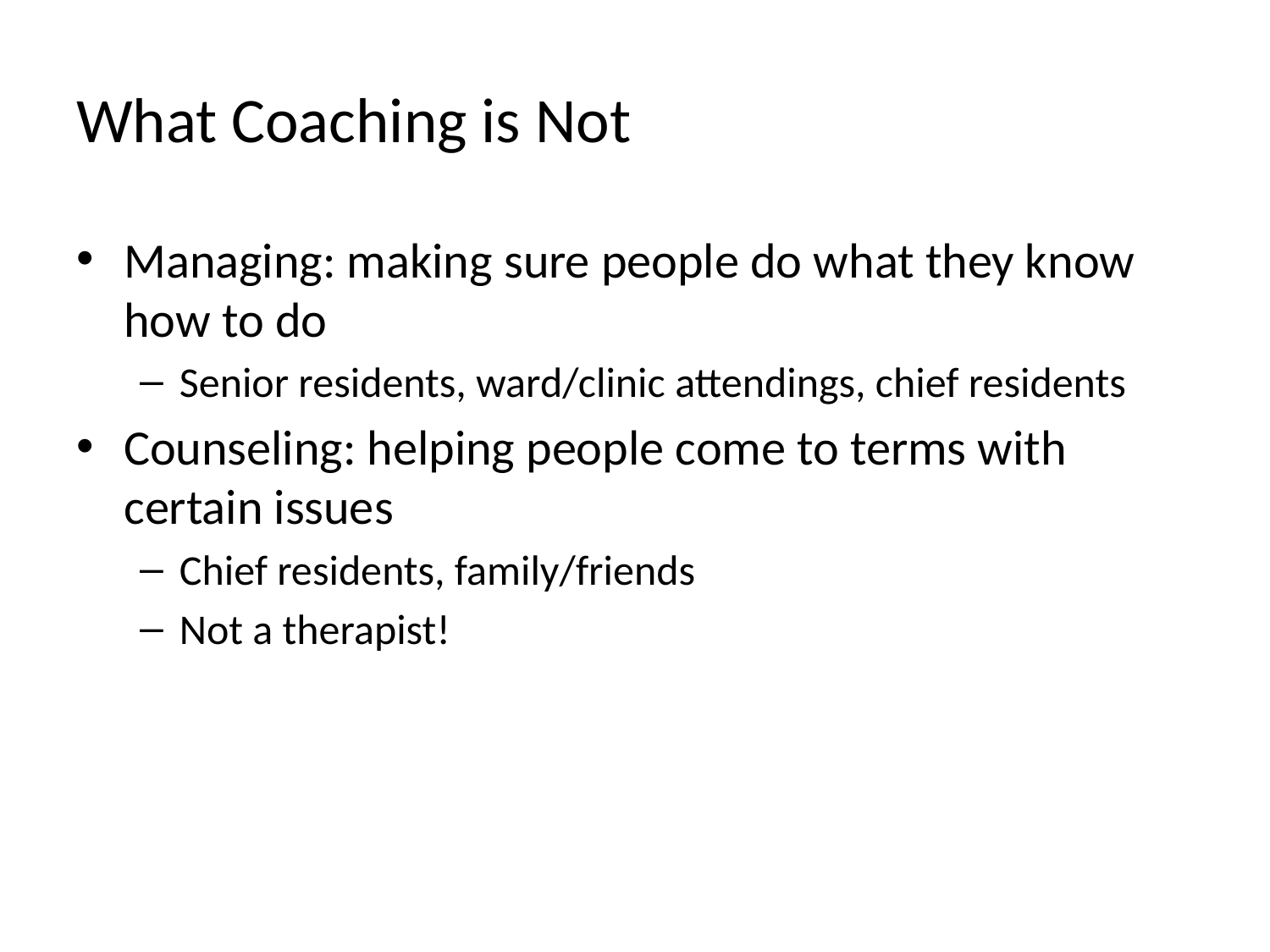

# What Coaching is Not
Managing: making sure people do what they know how to do
Senior residents, ward/clinic attendings, chief residents
Counseling: helping people come to terms with certain issues
Chief residents, family/friends
Not a therapist!

## Slide 32
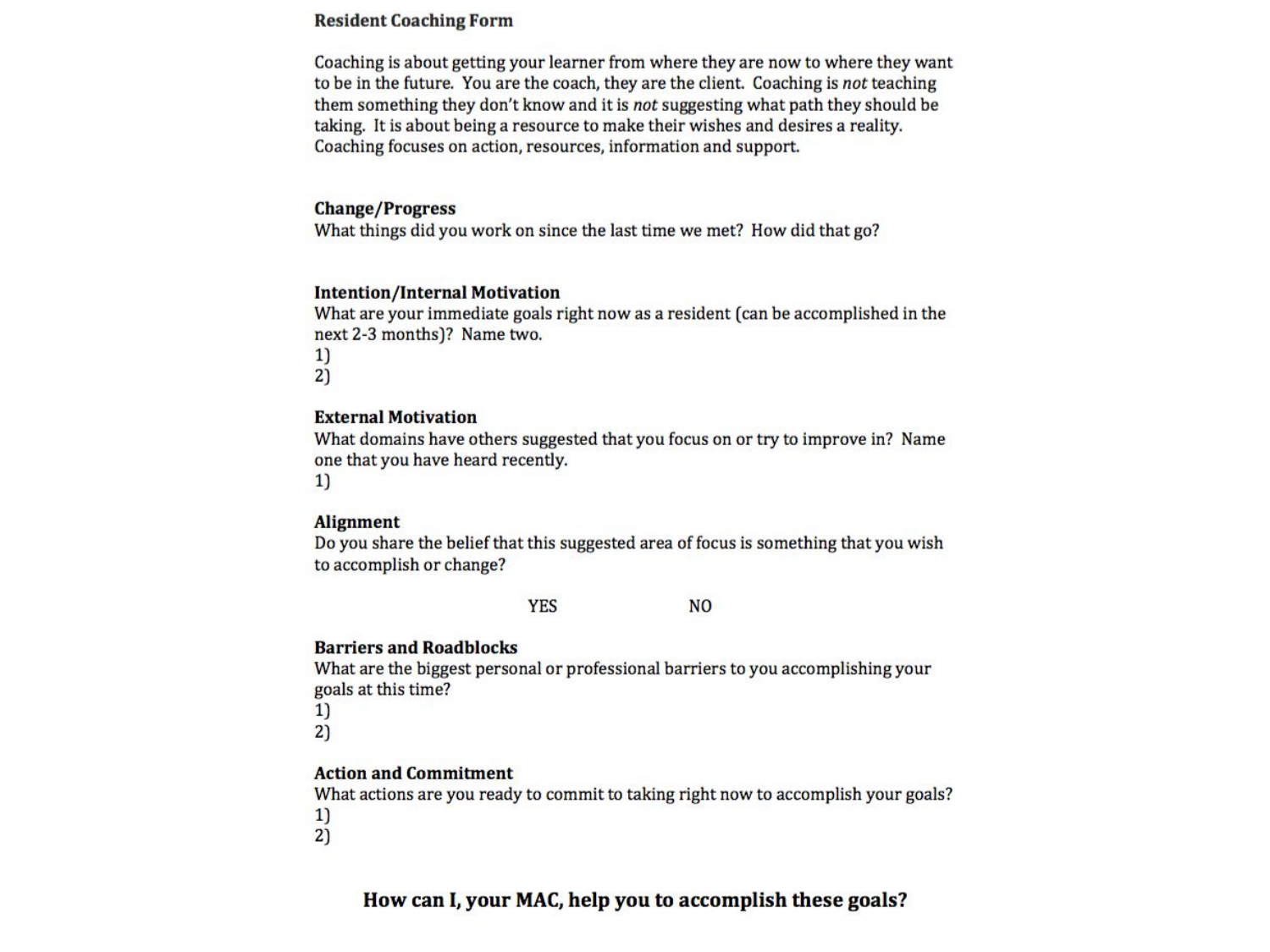

## Slide 33
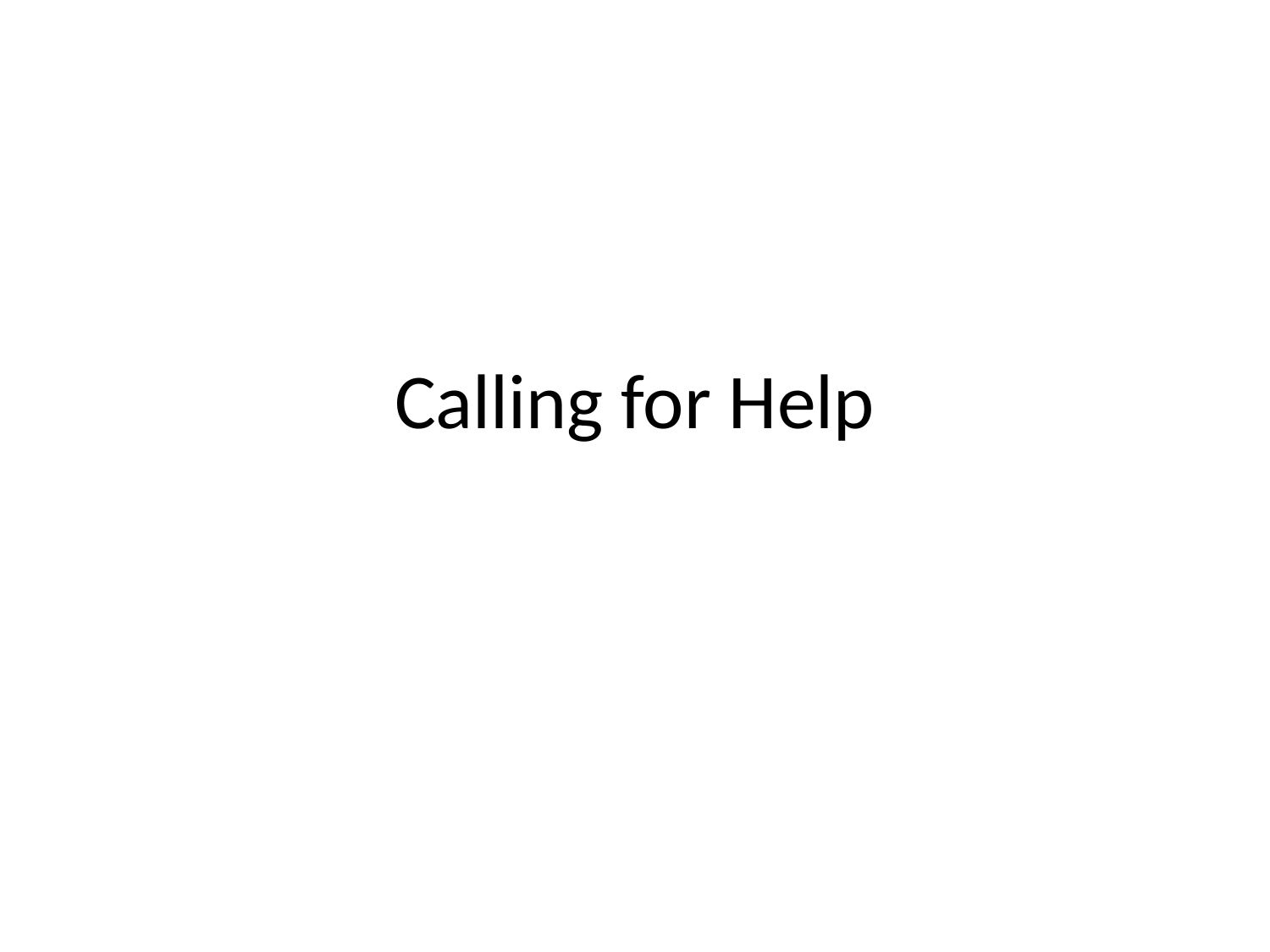

# Calling for Help

## Slide 34
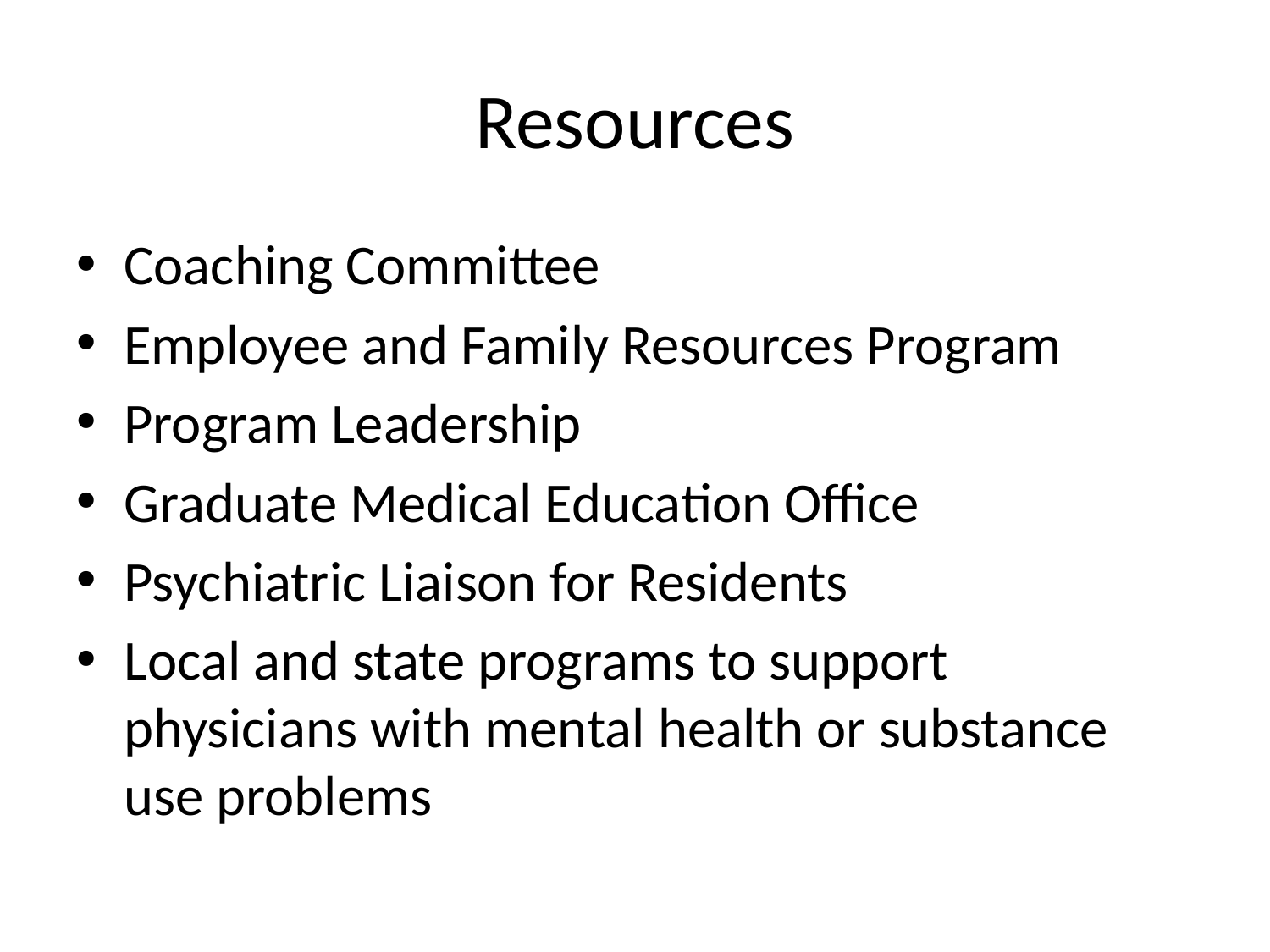

# Resources
Coaching Committee
Employee and Family Resources Program
Program Leadership
Graduate Medical Education Office
Psychiatric Liaison for Residents
Local and state programs to support physicians with mental health or substance use problems

## Slide 35
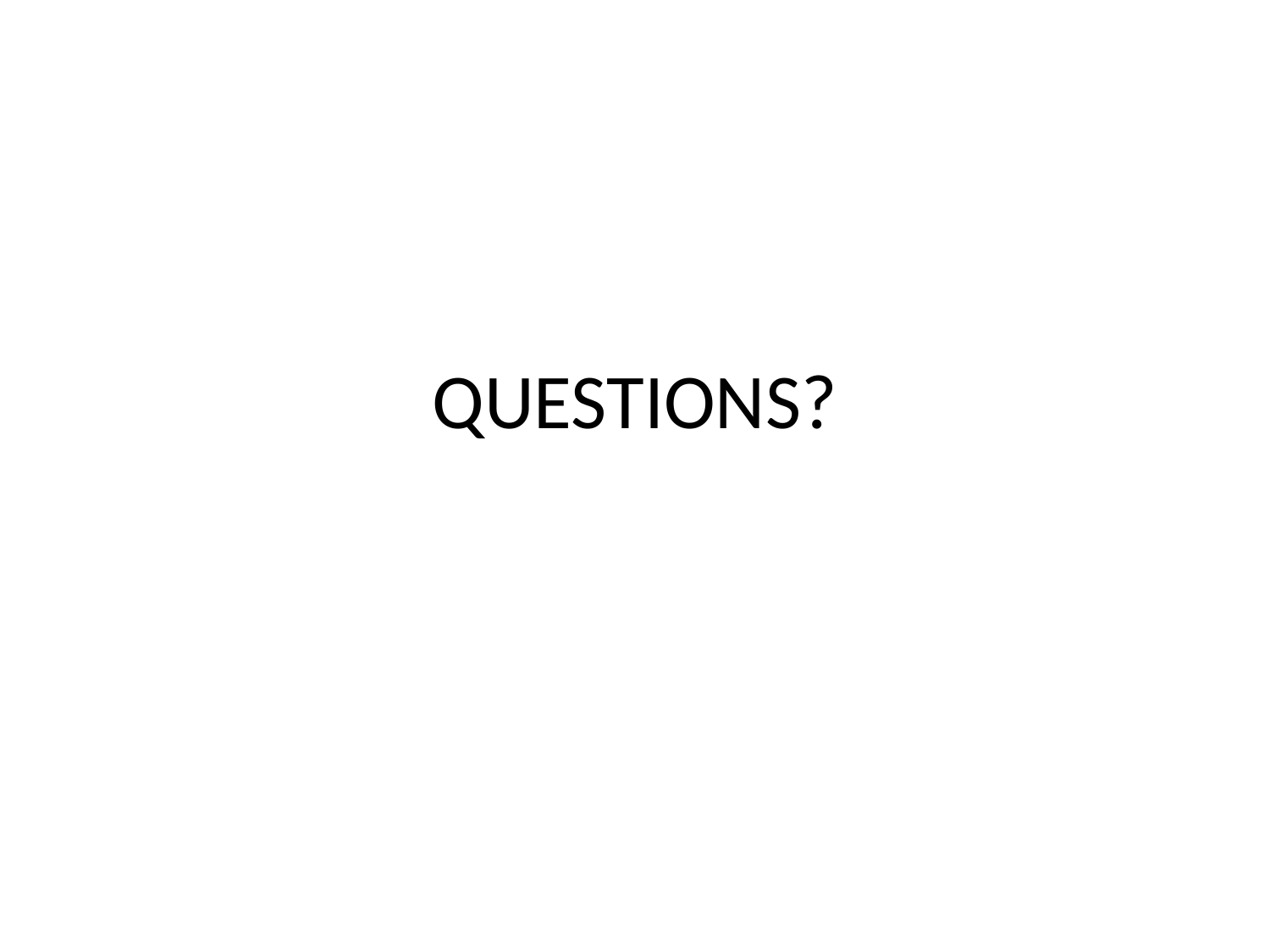

# QUESTIONS?
