# Supplementary material for: A Mentor, Advisor, and Coach (MAC) Program to Enhance the Resident and Mentor Experience
Source: MedEdPORTAL. 2020 Nov 3;16:11005. doi: 10.15766/mep_2374-8265.11005 (PMC7666835; doi:10.15766/mep_2374-8265.11005)
Supplement: Supplementary file 1 — MAC Training Presentation.pptxMAC Training Facilitator Guide.docxMAC Faculty Guide.docxMAC Survey - Resident Pairings.docxMeet and Greet Questionnaire.docCoaching Worksheet.docxMentoring Worksheet.docxQuestions for Focus Groups.docx [file mep_2374-8265.11005-s001.zip › B. MAC Training Facilitator Guide.docx]

**Guide to Implementing a MAC Program**

The Powerpoint slides have extensive notes to help you explain the program to your faculty and teach the key principles of coaching and mentoring. Please refer to the slides for the details of the program. The following is an overview of how to recruit, match and train your MACs.

Recruitment:

We suggest surveying your residents to identify faculty they feel would be good in the role. Once you have a list from the residents, the program leadership should vet the list. In our experience, more junior faculty tend to be able to establish better relationships with the residents and, since they are closer to the experience, more able to give sound advice about navigating residency. However, some of our best MACs have been more senior and, therefore, we would not exclude faculty solely based on rank or age. We excluded faculty who hold leadership positions within fellowship programs to avoid conflicts of interest. Make sure to think about how many faculty of various genders you will need, as we have found gender matching to be one of the most important factors for creating good relationships.

One of the most gratifying aspects of this process for us has been how easy it has been to recruit the faculty. Our faculty are enthusiastic to help residents despite not being able to offer them time or money to do so. We do emphasize the academic credit they will get for serving in this role, which has in recent years become a more important part of promotion at our institution.

Matching:

Prior to interns arriving, we survey them to find out what career fields they are interested in, what types of career they may want to pursue, their demographics (including where they grew up, went to college and medical school), and anything they feel is important for matching (we give them examples such as mentors with families, sexual orientation, gender, etc.) When matching, we use the list of faculty MACs to find someone of the same gender who is located physically where the intern’s clinic will be and then look at the factors the interns have listed as important. We also make sure to match AGAINST career field so that there is no conflict of interest that arises and so that the relationship can truly be a safe space for the residents. Matching is done by a group from program leadership so that there is someone who knows each faculty personally to aid in matching.

It is important to check in with the residents to see if the relationship is a successful one. We ask about the MAC relationship at each of the residents’ semi-annual meetings with program leadership. It occasionally becomes necessary to switch a resident to a new MAC, so it is important to warn the MACs that this may happen.

Training:

Faculty development for the MACs at our program includes two sessions/year. One session is the initial training on “How to Be a MAC” (slide show included) for new MACs. The other session is for all MACs, is different each year, and addresses a topic of interest and use to the MACs in their role. Examples include Fostering a Growth Mindset in Trainees and Self-Regulated Learning Theory.

Tips for a successful training session:

1. Serve food to encourage attendance, thank the MACs for their hard work, and create a more informal atmosphere to stimulate questions and discussion.
2. Consider developing local expertise in the topics addressed in the slideshow-- it is a great opportunity for someone in program leadership or on faculty to become more knowledgeable in one of the key aspects of the program. This expertise can be useful otherwise to the program leadership. Having more than one speaker giving the talk can also make it more interesting and engaging.
3. Take feedback from your MACs and residents about what works best for them. This program can be adapted easily for the needs of your residents and faculty. Set aside time at each faculty development session with the MACs for questions, discussions of the program broadly, and sharing of best practices. Some of the most useful teaching has come from the MACs themselves. Speak to your residents regularly or survey them to find out what they need from the program.
4. Solicit ideas for topics for the faculty development sessions from MACs. They will often have great thoughts about what they would like to learn more thoroughly.
