# Supplementary material for: A Mentor, Advisor, and Coach (MAC) Program to Enhance the Resident and Mentor Experience
Source: MedEdPORTAL. 2020 Nov 3;16:11005. doi: 10.15766/mep_2374-8265.11005 (PMC7666835; doi:10.15766/mep_2374-8265.11005)
Supplement: Supplementary file 1 — MAC Training Presentation.pptxMAC Training Facilitator Guide.docxMAC Faculty Guide.docxMAC Survey - Resident Pairings.docxMeet and Greet Questionnaire.docCoaching Worksheet.docxMentoring Worksheet.docxQuestions for Focus Groups.docx [file mep_2374-8265.11005-s001.zip › C. MAC Faculty Guide.docx]

Faculty Guide to Being a MAC

The MAC program was established to improve the support of our residents during their training. All residents can benefit from one on one mentorship and coaching. We are fortunate to have faculty who are invested in our residents’ development and who can provide that one on one support. Thank you for being a MAC-- you will make a big difference in our residents’ lives.

Principles of the MAC-Resident Relationship:

1. All conversations and aspects of the relationship are confidential unless:
   1. the resident agrees that the MAC can speak to program leadership about any concerns or questions regarding the resident OR
   2. there are any concerns by the MAC regarding patient or resident safety.

This confidentiality allows the residents to feel safe being honest with the MAC about insecurities, areas for improvement, confusion about career goals, etc.

1. The relationship should focus on what is important to the resident. The roadmap exists to provide structure to the program but can be deviated from as needed by the resident.
2. Active listening and non-judgmental support are key to the success of these relationships. Good mentoring relationships are ones which are resident driven, emotionally safe, supportive, respectful and informal, and where the mentor is responsive to the needs of the resident. These relationship foundations help to the resident to develop the independence, self-reflection and synthesizing skills to be successful.
3. We hope residents will feel free to be honest here in a way they may not feel they can be elsewhere in their training. Please try to listen openly and remember your role is not to fix them but to help guide them through whatever difficulties they may encounter.
4. We hope you as a MAC also gain a lot from this experience. There is enormous satisfaction in helping residents through what is likely to be the most challenging part of their careers.

Structure of the Program:

Each MAC is assigned up to one resident/class, for a potential total of three residents. Each resident class has a specific roadmap for the meetings with their MACs (see slideshow for roadmaps). The roadmaps outline what should be discussed at each meeting and we will provide supporting documents to make those meetings easier. We will email you when it is time to meet again with the topic of the meeting and the documents needed for the meeting. We will also email the residents, and they should reach out to you to set up the meeting. It also works nicely to set up your next meeting time at the time you meet. Remember that residents may want to deviate from the roadmap schedule if there is something of concern to them-- please allow them to do so but try to go back at some point and complete the meeting that was missed.

We recommend you try to meet during residents’ ambulatory blocks, which are 2 weeks long and happen every 2 months. They have more free time during ambulatory blocks, making meeting easier. We have assigned you residents whose clinic site matches up with where you work so that meeting will be easier physically as well. You can meet in your office, over coffee, for lunch, etc. Please make sure there is some privacy for the resident to feel they can discuss sensitive issues if needed.

Topics of Meetings:

1. Meet and Greet: an opportunity to get to know your resident. We have a questionnaire you can complete together to make sure you know about their career plans, if they have any at this point.
2. Coaching: Coaching is one of the most important and difficult parts of being a MAC. The slideshow contains a lot of information about coaching principles-- please review it if you have questions or feel free to reach out to program leadership for advice.
   1. Coaching involves allowing the resident to identify goals for improvement. You will help them identify these goals by reviewing their evaluations with them and looking for themes.
   2. The path to improvement should also be something the resident develops themselves although you will be invaluable to helping them do this. Help them think through how they might improve in the area identified (readings, watching videos, asking for direct observation and feedback from other residents, fellows or attendings) and when they will know if they have reached their goal (what does success look like?)
   3. Set up a clear action plan for their end goal (the overall improvement they seek) that includes means goals (the steps they will need to take to get there). For example, if your intern wants to improve their presentations (end goal), you can work together to identify steps to get there such as working with their resident to identify the sections of the history and physical that are key to present, practicing their presentations, and getting feedback on their presentations from the attending (means goals).
   4. The next coaching meeting will review how the action plan went and will also involve a review of evaluations again to see if improvement is noted in the evaluations. If the goal has not been met, you can discuss why not and come up with another action plan. If it has been met, great, time to set a new goal.
   5. This process will help residents learn how to self-improve throughout their career and is invaluable to their development
   6. If you are concerned that the coaching needed is beyond your ability or represents a serious deficit in the resident’s skills, please ask the resident if you can speak to us in program leadership so we can set up a more formal coaching plan.
3. Career: The career meeting is to help residents identify what they want to do and find mentors in that field. Many residents will be clear about their career plans early on in residency, and some will be unclear until the end. You can make suggestions for ways to gain experiences to help them choose a career field such as doing elective rotations, asking the chiefs to be put on specific rotations, asking for an outpatient clinic experience, attending journal clubs and grand rounds for that specialty, etc. Again, your path to choosing a career can be very helpful to them. When they have chosen a career field, also help them think of ways to find a mentor. Many of the experiences listed above can introduce them to faculty in that field. You may also help connect them with someone you think would be a good match based on interests and personality.
4. Wellness: The wellness meeting will take place in the dead of winter, when there is a proven dip in resident morale. Check in with your resident about how they try to stay well when tired and cooped up. Ask about their physical, spiritual (can take many forms, not just religion), and mental health. Brainstorm ways to stay healthy in those domains including regular exercise, attending worship services or meditation if that is important to them, and staying connected socially. Share how you coped during the dark days of residency and how you cope now. Your experiences will be very helpful to them.

If you have significant concerns about their mental health, please encourage them to seek help through the resources listed at the end of the slide show or, if you are worried about their or patient safety, please contact program leadership immediately.

The program will periodically provide information to you about your residents which will be helpful as you mentor them. As noted above, we will never ask you for information about your residents.

We are here to help you. Please feel free to reach out with generic questions about coaching, mentoring, etc, we can help you work through how best to support your residents.

Thank you again for your time and dedication to the MAC program. We and our residents deeply appreciate it!!
