# Supplementary material for: A Mentor, Advisor, and Coach (MAC) Program to Enhance the Resident and Mentor Experience
Source: MedEdPORTAL. 2020 Nov 3;16:11005. doi: 10.15766/mep_2374-8265.11005 (PMC7666835; doi:10.15766/mep_2374-8265.11005)
Supplement: Supplementary file 1 — MAC Training Presentation.pptxMAC Training Facilitator Guide.docxMAC Faculty Guide.docxMAC Survey - Resident Pairings.docxMeet and Greet Questionnaire.docCoaching Worksheet.docxMentoring Worksheet.docxQuestions for Focus Groups.docx [file mep_2374-8265.11005-s001.zip › D. MAC Survey - Resident Pairings.docx]

Welcome to the Internal Medicine Residency Program!  We are so excited for you to join our family.  In addition to our longstanding peer mentoring program, we will assign each of you to a faculty member who will serve as your MAC (Mentor/Advisor/Coach.)  You and your MAC will have regularly scheduled meetings during your ambulatory blocks throughout your time with us.  Your MAC will help you with career decision making and finding career and research mentors, and will help coach you to become the best physician you can be.

We want to be clear that your MAC **will not** be your primary career mentor and will actually try our best to match you with someone who does not work in the specialty you are interested in.  Because this person will be coaching you as well, it is imperative that they do not have any role in evaluating you during residency nor have any influence over whether you get a fellowship or job in your selected field. 

In order to match you as best we can, we ask that you complete the following brief survey.

**1. Name:**

*** 2. Medical School:**

*** 3. College and major:**

*** 4. Where did you grow up?**

*** 5. Do you want to be matched by any of the following demographic information? Choose all that apply.**

Gender

Race

Religion

Sexual orientation

None of these matter to me

Other (please specify)

*** 6. Do you want to be matched by any of the following? Choose all that apply.**

 Medical School

College

Hometown/region of the country/foreign country

None of these matter to me

Other (please specify)

*** 7. Do you know what your career interests are?**

Yes

No

If yes, what are your career interests?

*** 8. If you want to be matched based on career interests, please choose type of career below (skip this question if you don’t want to be matched based on career interests):**

Clinician-educator

Clinician

Physician-Scientist

I don't know what type of career I am interested in yet

Other:

*** 9. How certain are you of your choices?**

Very certain

Fairly certain

Somewhat certain

Not at all certain

*** 10. Please let us know if you have any further comments, requests or suggestions**
