# Supplementary material for: A Mentor, Advisor, and Coach (MAC) Program to Enhance the Resident and Mentor Experience
Source: MedEdPORTAL. 2020 Nov 3;16:11005. doi: 10.15766/mep_2374-8265.11005 (PMC7666835; doi:10.15766/mep_2374-8265.11005)
Supplement: Supplementary file 1 — MAC Training Presentation.pptxMAC Training Facilitator Guide.docxMAC Faculty Guide.docxMAC Survey - Resident Pairings.docxMeet and Greet Questionnaire.docCoaching Worksheet.docxMentoring Worksheet.docxQuestions for Focus Groups.docx [file mep_2374-8265.11005-s001.zip › F. Coaching Worksheet.docx]

**Coaching Worksheet**

Coaching is about getting your learner from where they are now to where they want to be in the future. You are the coach, they are the client. Coaching is *not* teaching them something they don’t know and it is *not* suggesting what path they should be taking. It is about being a resource to make their wishes and desires a reality. Coaching focuses on action, resources, information and support.

**Change/Progress**

What things did you work on since the last time we met? How did that go?

**Intention/Internal Motivation**

What are your immediate goals right now as a resident (can be accomplished in the next 2-3 months)? Name two.

1)

2)

**External Motivation**

What domains have others suggested that you focus on or try to improve in? Name one that you have heard recently.

1)

**Alignment**

Do you share the belief that this suggested area of focus is something that you wish to accomplish or change?

YES NO

**Barriers and Roadblocks**

What are the biggest personal or professional barriers to you accomplishing your goals at this time?

1)

2)

**Action and Commitment**

What actions are you ready to commit to taking right now to accomplish your goals?

1)

2)

**How can I, your MAC, help you to accomplish these goals?**
