# Supplementary material for: A Mentor, Advisor, and Coach (MAC) Program to Enhance the Resident and Mentor Experience
Source: MedEdPORTAL. 2020 Nov 3;16:11005. doi: 10.15766/mep_2374-8265.11005 (PMC7666835; doi:10.15766/mep_2374-8265.11005)
Supplement: Supplementary file 1 — MAC Training Presentation.pptxMAC Training Facilitator Guide.docxMAC Faculty Guide.docxMAC Survey - Resident Pairings.docxMeet and Greet Questionnaire.docCoaching Worksheet.docxMentoring Worksheet.docxQuestions for Focus Groups.docx [file mep_2374-8265.11005-s001.zip › G. Mentoring Worksheet.docx]

**Mentoring Worksheet:**

**Think about your career plan. What kind of mentoring do you need to get there?**

**What roles are unfilled?**

| Career Goal | What will be required to attain stated goal? | What type of mentoring will help? | Who might provide this?* | How and when will you reach out? |
| --- | --- | --- | --- | --- |
|  |  |  |  |  |
|  |  |  |  |  |
|  |  |  |  |  |

*If you don’t know of a person who could serve in this role, how will you identify a person to fill this role?

Mentoring Guidelines:

1. Be clear on why you want a mentor. Set clear objectives for the relationship.
2. When asking someone to be your mentor, explain why you’re asking and what you’d expect out of the relationship. Name your reasons for approaching this particular person.
3. A mentor is a powerful role model. Look for someone who has the kind of life and work you’d like to have. Also, choose a mentor you truly respect. Don’t just go for the biggest name you can find.
4. Show gratitude. Also, supply feedback. If your mentor suggested something that really worked out for you, report back. People love hearing about their part in a success story.
5. Keep in mind that mentoring can take many forms. It can be a monthly lunch, a quarterly phone call, or merely regular e-mail correspondence.
6. You’re allowed to have more than one mentor. In fact, you can have a whole committee if you want, and call it your Board of Directors. You can choose different mentors for different facets of your professional (and even personal) life.
7. Finally, if you ask someone to be your mentor and that person refuses, don’t be hurt or offended. This is not personal! Potential good mentors are very busy people. Thank him or her for the consideration, and ask for a referral.

(Adapted from John D Mahan, “The Professional Mission Statement: Defining Your Career Goals”; The Ohio State University College of Medicine and Karen Burns, “13 Tips on Finding a Mentor”; US News & World Report)
