# Supplementary material for: A Mentor, Advisor, and Coach (MAC) Program to Enhance the Resident and Mentor Experience
Source: MedEdPORTAL. 2020 Nov 3;16:11005. doi: 10.15766/mep_2374-8265.11005 (PMC7666835; doi:10.15766/mep_2374-8265.11005)
Supplement: Supplementary file 1 — MAC Training Presentation.pptxMAC Training Facilitator Guide.docxMAC Faculty Guide.docxMAC Survey - Resident Pairings.docxMeet and Greet Questionnaire.docCoaching Worksheet.docxMentoring Worksheet.docxQuestions for Focus Groups.docx [file mep_2374-8265.11005-s001.zip › H. Questions for Focus Groups.docx]

**Questions to guide focus group discussions**

**Focus groups with residents:**

1. How many times did you meet with your MAC faculty in one academic year?
2. Are there certain topics that you would be most interested in speaking to a MAC faculty about? What aspect of a relationship are you most interested in of the mentoring/advising/coaching realms?
3. What have you gained from your relationship with your MAC faculty?
4. Do you have other suggestions for improvements to the MAC program, or to the mentorship structure of our residency program?

**Focus groups with MAC faculty:**

1. Regarding the components of being a MAC faculty (mentor, advisor, coach):
   1. What are your thoughts on the three components?
   2. Do you understand of each component? Would you request further information on each component?
   3. What have the most valuable experiences been for you or your mentees?
   4. Should we focus more or less on any of these aspects?
2. How do you feel about being able to access evaluations of residents?
3. Regarding preparedness to answer questions on fellowship and job applications:
   1. What have mentees asked you for help with?
   2. What do you feel your role should be?
4. How can the Internal Medicine program leadership help you prepare for these questions?
