# Supplementary material for: A Mentor, Advisor, and Coach (MAC) Program to Enhance the Resident and Mentor Experience
Source: MedEdPORTAL. 2020 Nov 3;16:11005. doi: 10.15766/mep_2374-8265.11005 (PMC7666835; doi:10.15766/mep_2374-8265.11005)
Supplement: Supplementary file 1 — MAC Training Presentation.pptxMAC Training Facilitator Guide.docxMAC Faculty Guide.docxMAC Survey - Resident Pairings.docxMeet and Greet Questionnaire.docCoaching Worksheet.docxMentoring Worksheet.docxQuestions for Focus Groups.docx [file mep_2374-8265.11005-s001.zip › E. Meet and Greet Questionnaire.doc]

**Meet and Greet Questionnaire**

Name______________________________________________

Medical School­­­­­­­­­­­______________________________________

College_____________________________________________

Where did you grow up?________________________________

1. Do you have a sense of what type of career you’d like to have (academic, research, education, private practice, industry?)

2. What are your outside interests (music, arts, sports…)?

3. Are you interested in finding a faculty mentor? Yes	No

**(For second year residents)**

If so, do you have someone in mind?

Who?_________________________________

Have you already met with or contacted this person? Yes	No

4. Are there any areas that you would like some extra help with (knowledge/skills/attitudes)?
